# A Conserved Endoplasmic Reticulum Membrane Protein Complex (EMC) Facilitates Phospholipid Transfer from the ER to Mitochondria

**DOI:** 10.1371/journal.pbio.1001969

**Published:** 2014-10-14

**Authors:** Sujoy Lahiri, Jesse T. Chao, Shabnam Tavassoli, Andrew K. O. Wong, Vineet Choudhary, Barry P. Young, Christopher J. R. Loewen, William A. Prinz

**Affiliations:** 1 Laboratory of Cell and Molecular Biology, National Institute of Diabetes and Digestive and Kidney Diseases, National Institutes of Health, Bethesda, Maryland, United States of America; 2 Department of Cellular and Physiological Sciences, Life Sciences Institute, University of British Columbia, Vancouver, British Columbia, Canada; UT Southwestern Medical Center, United States of America

## Abstract

Mitochondrial membrane biogenesis and lipid metabolism require phospholipid transfer from the endoplasmic reticulum (ER) to mitochondria. Transfer is thought to occur at regions of close contact of these organelles and to be nonvesicular, but the mechanism is not known. Here we used a novel genetic screen in *S. cerevisiae* to identify mutants with defects in lipid exchange between the ER and mitochondria. We show that a strain missing multiple components of the conserved ER membrane protein complex (EMC) has decreased phosphatidylserine (PS) transfer from the ER to mitochondria. Mitochondria from this strain have significantly reduced levels of PS and its derivative phosphatidylethanolamine (PE). Cells lacking EMC proteins and the ER–mitochondria tethering complex called ERMES (the ER–mitochondria encounter structure) are inviable, suggesting that the EMC also functions as a tether. These defects are corrected by expression of an engineered ER–mitochondrial tethering protein that artificially tethers the ER to mitochondria. EMC mutants have a significant reduction in the amount of ER tethered to mitochondria even though ERMES remained intact in these mutants, suggesting that the EMC performs an additional tethering function to ERMES. We find that all Emc proteins interact with the mitochondrial translocase of the outer membrane (TOM) complex protein Tom5 and this interaction is important for PS transfer and cell growth, suggesting that the EMC forms a tether by associating with the TOM complex. Together, our findings support that the EMC tethers ER to mitochondria, which is required for phospholipid synthesis and cell growth.

## Abstract 2

Tethering of the endoplasmic reticulum to mitochondria by a conserved endoplasmic reticulum complex is needed for the transfer of phospholipids between these organelles.

## Author Summary

Mitochondrial membrane biogenesis and lipid metabolism depend on the transfer of phospholipid from the endoplasmic reticulum to mitochondria. This transfer is thought to occur at regions where these organelles are in close contact, and, although the process is thought not to involve vesicles, the mechanism is not known. In this study, we found a complex of proteins in the endoplasmic reticulum that is required for the transfer of one phospholipid—phosphatidylserine—from the endoplasmic reticulum to mitochondria. Cells lacking this protein complex have nonfunctional mitochondria with an abnormal lipid composition. We show that the complex is required to maintain close contacts between the endoplasmic reticulum and mitochondria; the complex probably directly interacts with at least one protein on the surface of mitochondria. In addition, cells lacking this complex and a second previously identified tethering complex are not viable. Thus, our findings suggest that tethering of the endoplasmic reticulum and mitochondria is essential for cell growth, likely because it is necessary for lipid exchange between these organelles.

## Introduction

Mitochondria are critical cellular components that are needed for energy production, lipid metabolism, calcium regulation, and apoptosis. Most proteins and lipids necessary for mitochondrial biogenesis are not synthesized in mitochondria and must be imported. Although protein import into mitochondria is relatively well understood, much less is known about phospholipid transfer to mitochondria. Phospholipid synthesis occurs largely in the endoplasmic reticulum (ER), and mitochondria acquire phospholipids from the ER at regions of close contact between these organelles [1]–[3]. Zones of close contact between organelles, often called membrane contact sites, are regions where lipids, small molecules, and other signals are transferred between organelles [4]–[6]. Contacts between the ER and mitochondria are not only important for lipid exchange and signaling between these organelles, but have also been proposed to play a role in calcium signaling, apoptosis, Alzheimer's disease pathology, and viral replication [7]–[10].

Protein complexes proposed to mediate ER–mitochondria contacts have been identified in mammalian cells and in *Saccharomyces cerevisiae* [11]–[14]. The only such complex that has been found in yeast to date is called the ER–mitochondria encounter structure (ERMES), which comprises the integral ER glycoprotein, Mmm1, the cytosolic protein, Mdm12, and two proteins of the outer mitochondrial membrane, Mdm10 and Mdm34 [14]. The ERMES complex may play a role in phospholipid exchange between the ER and mitochondria. Notably, three of the four proteins contain a domain that may facilitate lipid exchange between closely apposed membranes [15]. The study that identified the ERMES complex found that phospholipid exchange between the ER and mitochondria decreases 2–5-fold in cells missing this complex [14]. However, two subsequent studies found that the transfer of phosphatidylserine (PS) from the ER, where it is produced [16], to mitochondria, did not significantly slow in cells lacking the ERMES complex [17],[18]. These findings suggest that protein complexes in addition to ERMES mediate ER–mitochondria tethering, as lipid exchange between these organelles probably occurs only at contacts. In addition, the fact that cells lacking ERMES are viable suggests there may be other tethering complexes, as ER–mitochondria tethering is probably essential [19].

The mechanism of phospholipid exchange between the ER and mitochondria at sites of contact between these organelles is not well understood, but is thought to be nonvesicular [2],[3]. A small number of mutants that reduce ER to mitochondria lipid transfer have been identified. One is missing Met30, a subunit of an ubiquitin ligase complex [20] that ubiquitinates the transcription factor Met4, which in turn regulates PS transfer to mitochondria [21]. Cells missing both the ERMES complex and proteins needed to shape the ER have a similar ∼50% decrease in ER to mitochondria PS transfer [18]. However, why lipid exchange slows in these mutants is not understood.

**Figure 1:**
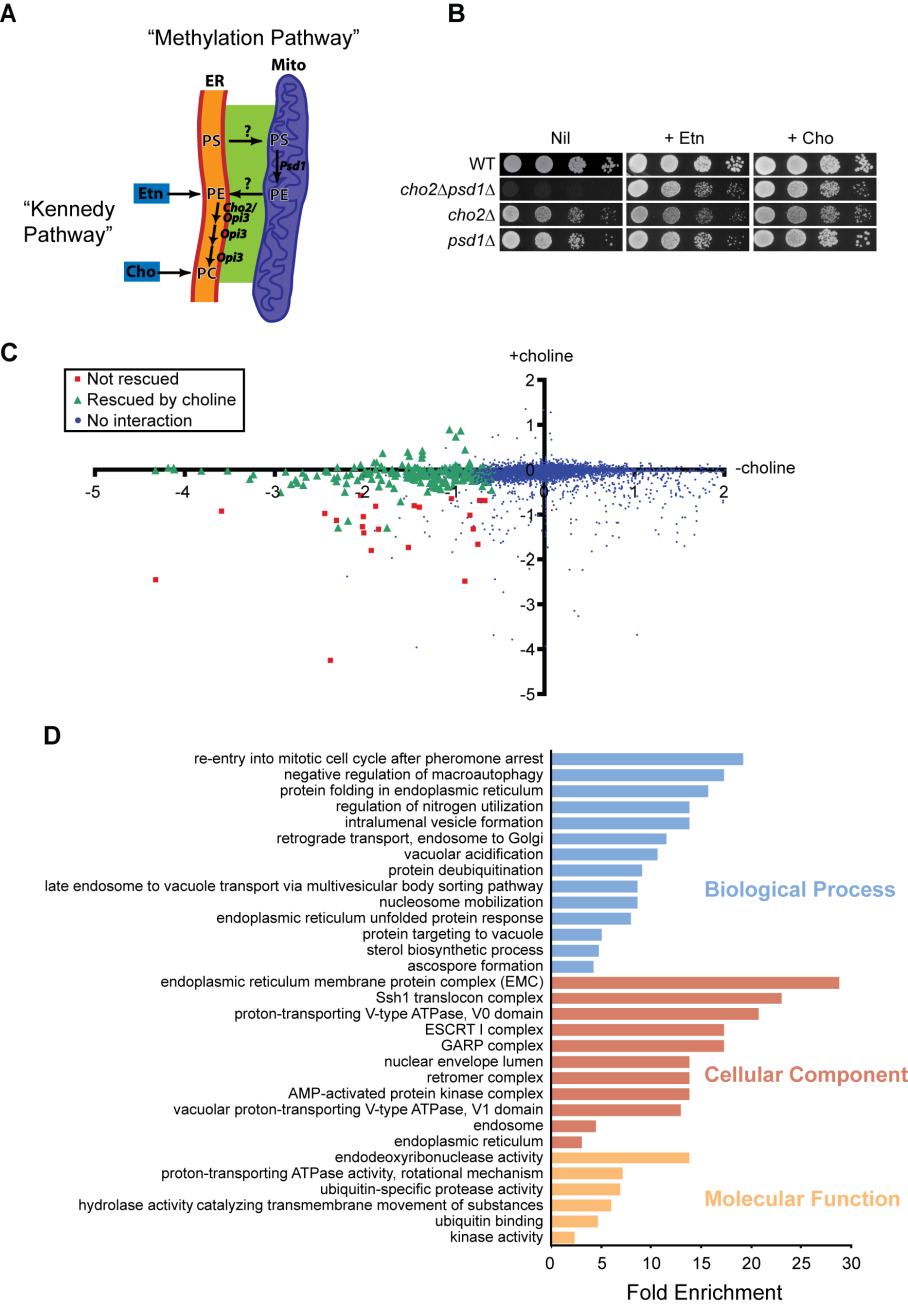
Genome-wide screen for regulators of phospholipid synthesis. (A) Phospholipid synthesis in the methylation pathway is compartmentalized between ER and mitochondria. PS synthesized in the ER is transferred to mitochondria for conversion into PE and transported back to the ER for conversion to PC. The Kennedy pathway synthesizes PE and PC from ethanolamine (etn) and choline (cho) independent of lipid transfer between ER and mitochondria. (B) Yeast growth assays for the indicated mutants in the absence (nil) or presence of ethanolamine (+ etn) or choline (+ cho). (C) Results of SGA screen for *CHO2* in the absence (–) and presence (+) of choline. Genetic interactions are plotted as the log2 of the ratio of growth of single versus double mutants with *Δcho2* in the absence and presence of choline. Interactions rescued by choline (green triangles) predominately clustered on the x axis, whereas interactions not rescued (red squares) were present on the diagonal. (D) Enrichment of functional groups for the genes that showed interactions and were rescued by choline in (C). Fold enrichment represents the frequency of a given term in our dataset relative to the frequency of that term in the whole genome.

PS transfer to mitochondria is required for the synthesis of phosphatidylethanolamine (PE) in mitochondria [2]. PE is critical for mitochondrial function [22],[23]. Although PE can be made outside mitochondria, for unknown reasons this PE is not efficiently transferred to mitochondria [24]. Failure to import PE may explain why all cells from yeast to humans have an enzyme that converts PS to PE in the mitochondrial matrix. In yeast, this protein is called PS decarboxylase 1 or Psd1 (Figure 1A) [25],[26]. PE produced in mitochondria by Psd1 can be transferred back to the ER and converted to phosphatidylcholine (PC) by the methyltransferases, Cho2 and Opi3 (Figure 1A). There is a second PS decarboxylase in yeast, called Psd2, which may reside in the Golgi complex, endosomal system, or vacuole [27]. PE can also be synthesized from diacylglycerol (DAG) and CDP–ethanolamine, a metabolic pathway known as the Kennedy pathway (Figure 1A) [28]. The Kennedy pathway can also produce PC from DAG and CDP–choline (Figure 1A).

In this study, we employed an array-based genetic interaction screen to identify genes required for phospholipid exchange between the ER and mitochondria. We found that mutants missing multiple proteins of the ER-membrane protein complex (EMC) had defects in PS transfer to mitochondria, reduced ER–mitochondrial tethering, and impaired mitochondria function. This complex contains six conserved proteins, called Emc1–6 [29]. The EMC has been suggested to play roles in the cellular response to ER stress, in membrane protein folding, or the unfolded protein response (UPR) in the ER [29]–[33]. However, the molecular functions of the EMC are not known. Our findings indicate that the EMC mediates lipid transfer from the ER to mitochondria by facilitating tethering between these organelles.

## Results

### Genetic Screen for Components That Mediate Transfer of Phospholipids Between ER and Mitochondria

In a prior synthetic genetic array (SGA) screen for the *PSD1* gene, we uncovered an aggravating genetic interaction with the *CHO2* gene. We found that the growth defect of the *Δpsd1Δcho2* mutant was rescued by the addition of ethanolamine or choline to the medium (Figure 1B), which allows cells to make PE and PC via the Kennedy pathway, indicating that this mutant had a defect in PE synthesis from PS (Figure 1A). We reasoned that genes required for transfer of PS from ER to mitochondria and PE from mitochondria to ER would similarly have negative genetic interactions with *CHO2* that would be rescued by ethanolamine or choline. Therefore, we performed SGA screens for the *CHO2* gene in the absence and presence of choline to identify genes that functioned in lipid transfer between ER and mitochondria. The results of this genome-wide screen are shown in Figure 1C, in which we plotted the growth of double mutants in the absence of choline versus their growth in its presence. We identified 209 aggravating genetic interactions in total, 187 that we deemed were rescued by choline addition (Figure 1C and Table S1). We found significant enrichment for various functional classifications for this set of genes, and these are shown in Figure 1D (see also Table S2). Membrane-associated functions were highly represented among these groups and included ER, vacuole, and endosomal compartments.

### Genetic Interactions Reveal That the EMC Functions in Phospholipid Synthesis

**Figure 2:**
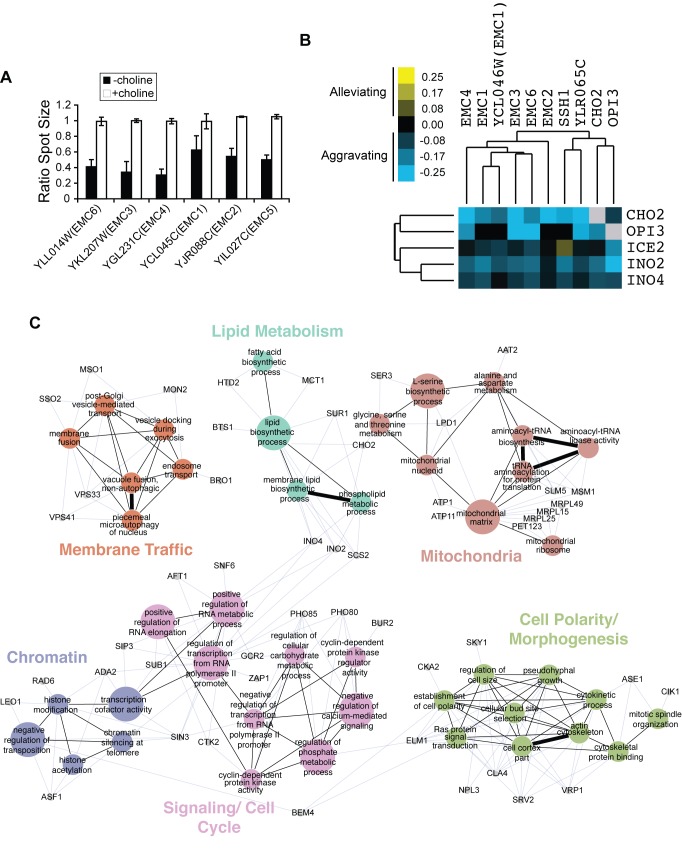
EMC genes function in phospholipid metabolism. (A) Genetic interactions identified between EMC genes and *CHO2*. Plotted is the ratio of spot size of the single EMC mutants versus the corresponding double mutants with *Δcho2* in the absence and presence of choline. The data used to generate this graph are in Table S1. (B) EMC gene cluster identified in the global genetic interaction map [34] and aggravating genetic interactions with a cluster of genes that function in the methylation pathway of phospholipid synthesis. Aggravating interactions have negative values, and alleviating interactions have positive values. Trees identify isolated clusters identified in the global genetic interaction map. The data used to generate this figure have been published [34]. (C) Functional map for *EMC6* derived using genetic interactions identified in the *EMC6* SGA screen. Colored nodes represent functional groups, and edges define associations between groups. Node size and edge thickness indicate their level of significance within the network. Genes (grey nodes) identified in the screen that are associated with each functional group are shown (blue edges).

In the Cellular Component category, we noticed an almost 30-fold enrichment for subunits of the EMC, a conserved uncharacterized ER membrane protein complex (Figure 1D) [29]. In our screen, genes encoding the six subunits of the EMC showed strong aggravating genetic interactions with *CHO2* that were rescued by choline addition (Figure 2A). We verified these genetic interactions and their rescue by choline by spot assay (Figure S1). Interestingly, these interactions were not rescued by ethanolamine, suggesting that the EMC has functions distinct from PE production by Psd1.

To uncover additional functional information about the EMC, we examined the global genetic interaction network, which is a comprehensive map of pairwise genetic interactions in which genes with similar functions form coherent clusters [34]. We noticed that all EMC genes except for *EMC5*, which was not present in the global network, formed a discrete cluster, suggesting EMC genes share similar functions (Figure 2B). Interestingly, also in this cluster were *CHO2* and *OPI3*, which encode the two methyltransferases that convert PE to PC (Figure 1A), supporting a role for EMC genes in phospholipid metabolism. EMC genes showed primarily aggravating genetic interactions with a cluster of genes with roles in lipid metabolism including *ICE2*, *INO2*, and *INO4.* The latter two encode both subunits of the Ino2/4 transcriptional activator complex required for expression of genes involved in phospholipid synthesis, including *OPI3*.

Next, we performed an SGA screen for one of the EMC genes, *EMC6*, to further define functions for the EMC complex. We identified 37 aggravating and 45 alleviating genetic interactions with *EMC6* (Table S3). Genetic interactions with *EMC6* revealed enrichment for functions associated with lipid metabolism, mitochondria, membrane traffic, cell polarity and morphogenesis, cell signaling, and chromatin (Figure 2C). Although EMC genes have been found to have links to the UPR [29]–[31], we did not find significant enrichment for ER stress response functions and *EMC6* did not interact genetically with either *HAC1* or *IRE1*, two key factors required for induction of the UPR. Our screen did uncover genetic interactions with key regulators of phospholipid metabolism, *INO2*, *INO4*, and *SCS2*, as well as *CHO2* (Figure 2C), further supporting a role for EMC proteins in phospholipid synthesis. We also did not observe aggravating genetic interactions between *EMC6* and any of the remaining EMC genes (Table S3).

### EMC Proteins Form a Complex in the ER

**Figure 3:**
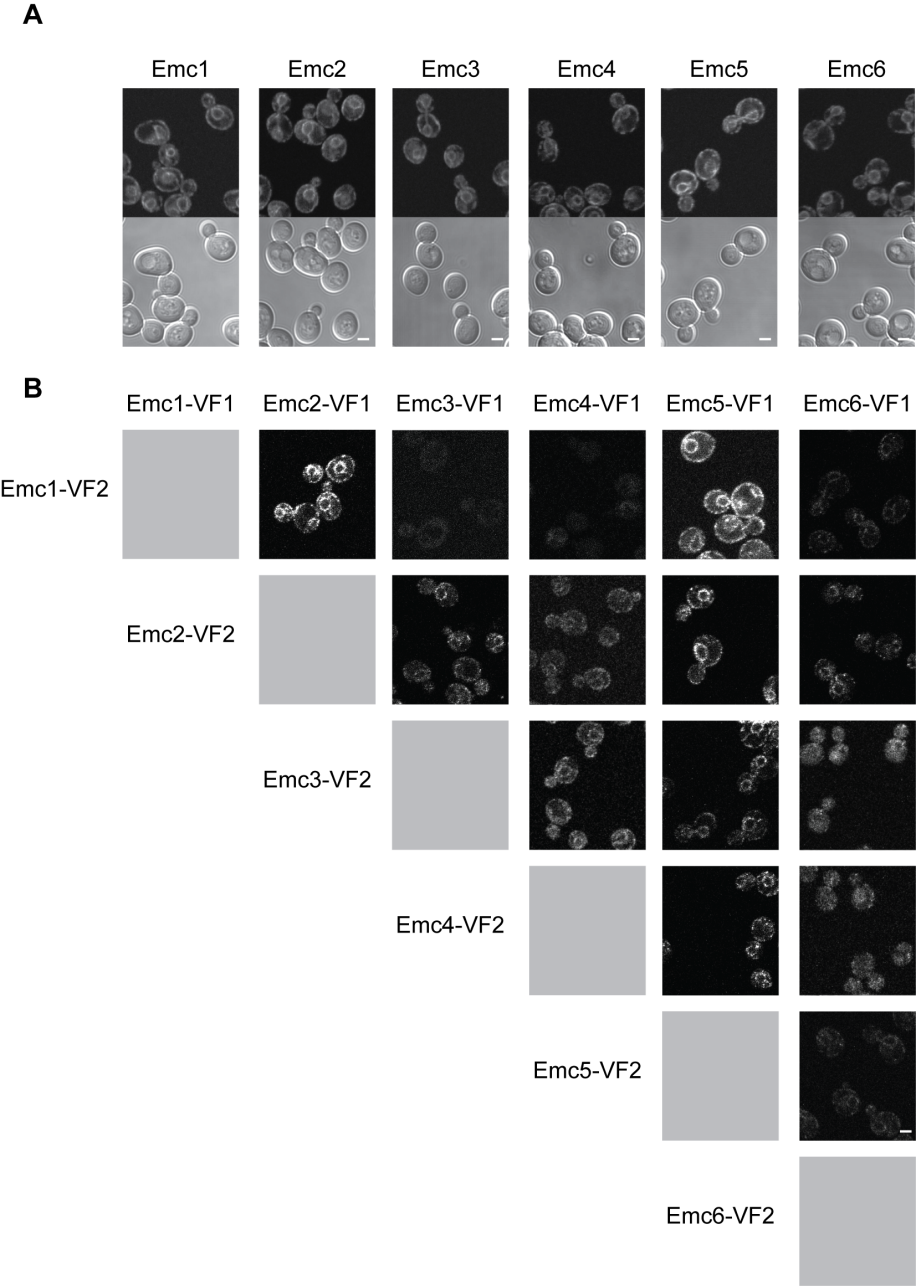
EMC proteins form a complex in the ER. (A) Yeast expressing EMC proteins endogenously tagged with GFP imaged by confocal microscopy; top panel, fluorescence image; bottom panel, DIC. (B) Interactions between EMC proteins in the ER imaged using Venus PCA. Images of cells expressing proteins fused to either of the two halves of the Venus proteins (VF1 or VF2). Scale bars, 2 µm.

EMC proteins 1–6 were first identified by their ability to interact in an affinity purification experiment [29]. We now examined their individual localizations by tagging the endogenous proteins with GFP and imaging by confocal microscopy. We found that each of Emc1–6 localized throughout the yeast ER and were expressed at similar levels (Figure 3A). Next we verified interactions between each EMC protein by Protein-Fragment Complementation Assay (PCA) in which we tagged each endogenous Emc protein with one half of the Venus fluorescent protein. When two proteins interact, the two halves of Venus bind each other (probably irreversibly) and form a fluorescent protein. Shown in Figure 3B is the matrix containing all pair-wise interactions between the EMC proteins. We observed PCA interactions in the ER between most EMC proteins, and there was no single EMC protein that failed to interact with any other EMC protein, suggesting that all six EMC proteins did indeed form a complex within the ER. Interestingly, we did not detect interactions between Emc1 and Emc3 or Emc1 and Emc4, whereas all other EMC proteins interacted, suggesting that Emc1 was organized distinctly within the complex.

### ER to Mitochondria PS Transfer Decreases in Cells Missing Multiple EMC Proteins

**Figure 4:**
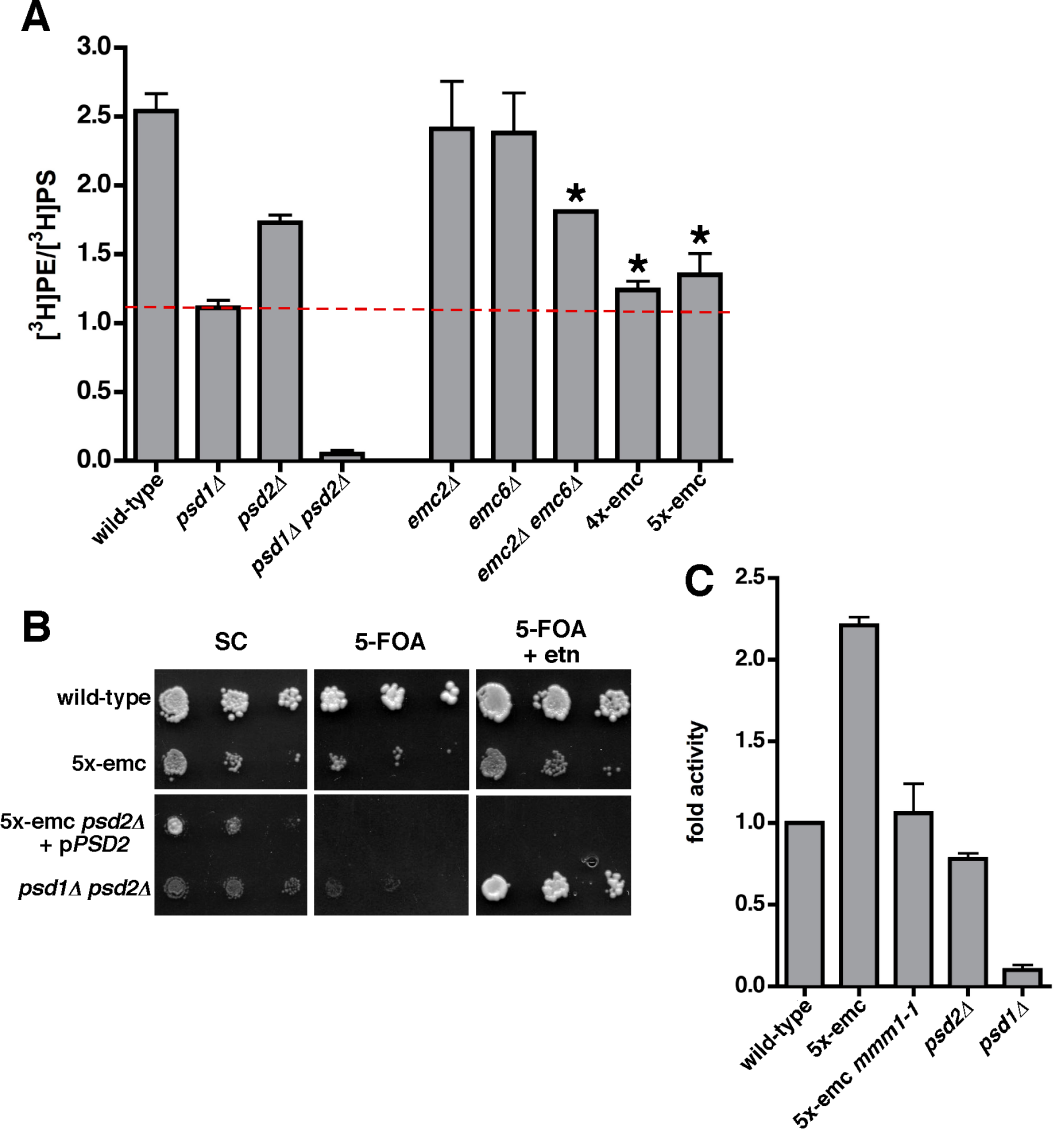
Cells missing multiple EMC proteins have defects in PS transfer from the ER to mitochondria. (A) Cells with the indicated genotypes were labeled with [^3^H]serine for 30 min and the ratio of [^3^H]PS converted to [^3^H]PE determined (mean ±s.d., *n* = 3–5 independent experiments). The dashed red line indicates the amount of conversion that occurred in *psd1*Δ cells. * *p*<0.05 compared to wild-type, independent two-tailed *t* test. (B) The 10-fold serial dilutions of cultures of the indicated strains were spotted onto SC medium with or without 5-FOA and ethanolamine. The plates were incubated at 30°C for 4 d. (C) PSD activity of crude mitochondria incubated with NBD-PS for 1 h at 30°C. PSD activity was normalized to that of wild-type crude-mitochondria (mean ±s.d., *n* = 2–3 independent experiments). * *p*<0.05 compared to wild-type, independent two-tailed *t* test. The data used to generate panels A and C are in Table S4.

To determine if the EMC plays a role in phospholipid exchange between the ER and mitochondria, we used an in vivo assay to measure PS import into mitochondria from the ER. After synthesis in the ER, PS can be transferred to mitochondria and converted to PE by Psd1 [28]. Thus, the conversion of newly synthesized PS to PE has been used to estimate the amount of PS transfer from the ER to mitochondria [21]. We metabolically labeled cells with [^3^H]serine, which is used for PS production in the ER [16]. Previously, we have shown that using the labeling conditions described in Materials and Methods, cells produce PS and PE at linear rates and that little of the radiolabeled PE is converted to PC [35]. Strains were labeled with [^3^H]serine for 30 min and the ratio of [^3^H]PE to [^3^H]PS calculated. In a wild-type strain, this ratio was 2.5. Cells missing either Psd1 or Psd2 had a significant decrease in the [^3^H]PE to [^3^H]PS ratio, and this ratio was close to zero in a strain missing both proteins (Figure 4A).

Using this assay we determined the amount of PS converted to PE in cells missing any one of the Emc proteins. We found that the amount of PS converted to PE did not decrease significantly in these strains (Figure 4A). However, in cells missing multiple EMC proteins, PS to PE conversion was significantly slowed. A strain lacking Emc2 and Emc6 had a ∼25% decrease in the ratio of [^3^H]PE to [^3^H]PS compared to wild-type. Cells missing additional Emc proteins had more substantial transfer defects; a strain missing Emc1, Emc2, Emc3, and Emc6 (4x-emc) or one missing these proteins and Emc5 (5x-emc) had ∼50% reduction in the ratio of [^3^H]PE to [^3^H]PS (Figure 4A). These strains contain Psd2, which is outside mitochondria and converts a significant fraction of newly synthesized PS to PE (Figure 4A). Because the amount of PS to PE conversion in 4x-emc and 5x-emc cells was about the same as that of cells lacking Psd1, our findings suggest that very little PS to PE conversion occurs in the mitochondria of these strains.

To rule out that the decrease in PS to PE conversion in 5x-emc cells was caused by a reduction in Psd1 activity or mislocalization of Psd1, we determined the amount of Psd activity in mitochondria derived from 5x-emc cells. An in vitro Psd assay was performed using a fluorescent PS analog [7-nitro-2–1,3-benzoxadiazol-4-yl]-PS (NBD-PS). We found mitochondrial Psd activity was not reduced in mitochondria from 5x-emc cells compared to those from wild-type cells, but rather was significantly increased, for unknown reasons (Figure 4C). In addition, we ruled out that the decrease in PS to PE conversion in 5x-emc cells was due to differences in total PS production during the labeling; there was no significant difference between total PS produced in wild-type and the EMC mutants (Figure S2A). We also confirmed that the rates of PS and PE production were linear in wild-type [35] and 5x-emc cells over the course of the 30-min labeling (Figure S2B). These findings confirm that 5x-emc cells have a significant decrease in the transfer of PS from the ER to mitochondria.

To test whether most of the PS converted to PE in 5x-emc cells was due to Psd2, we sought to make a 5x-emc *psd2*Δ strain and hence to measure PS to PE conversion only in the mitochondrial pathway. However, we found that 5x-emc *psd2*Δ cells were not viable. We transformed 5x-emc cells with a plasmid containing *PSD2* and *URA3* and then deleted *PSD2* on the chromosome. The resulting strain was not able to grow on medium with 5-fluoroorotic acid (5-FOA), which is toxic to *URA3* strains and selects against the plasmid carrying the *URA3* and *PSD2* genes (Figure 4B), confirming that the 5x-emc *psd2*Δ mutant was not viable. Adding ethanolamine to the medium, which is used to make PE by the Kennedy pathway (Figure 1A), and which restored viability of *psd1Δ psd2Δ* cells, did not rescue the 5x-emc *psd2*Δ mutant (Figure 4B). This suggests that the 5x-emc mutant might have lipid metabolism defects in addition to significantly reduced PS transfer to mitochondria. For example, it is possible that PE synthesis by the Kennedy pathway or the conversion of PE to PC is defective in 5x-emc cells. However, we found that both processes occurred at similar rates in wild-type and 5x-emc cells (Figure S3). Therefore, it remains unclear why 5x-emc *psd2*Δ cells are not viable even when grown in media containing ethanolamine.

### Mitochondria in 5x-emc Cells Are Nonfunctional and Have Abnormal Phospholipid Levels

**Figure 5:**
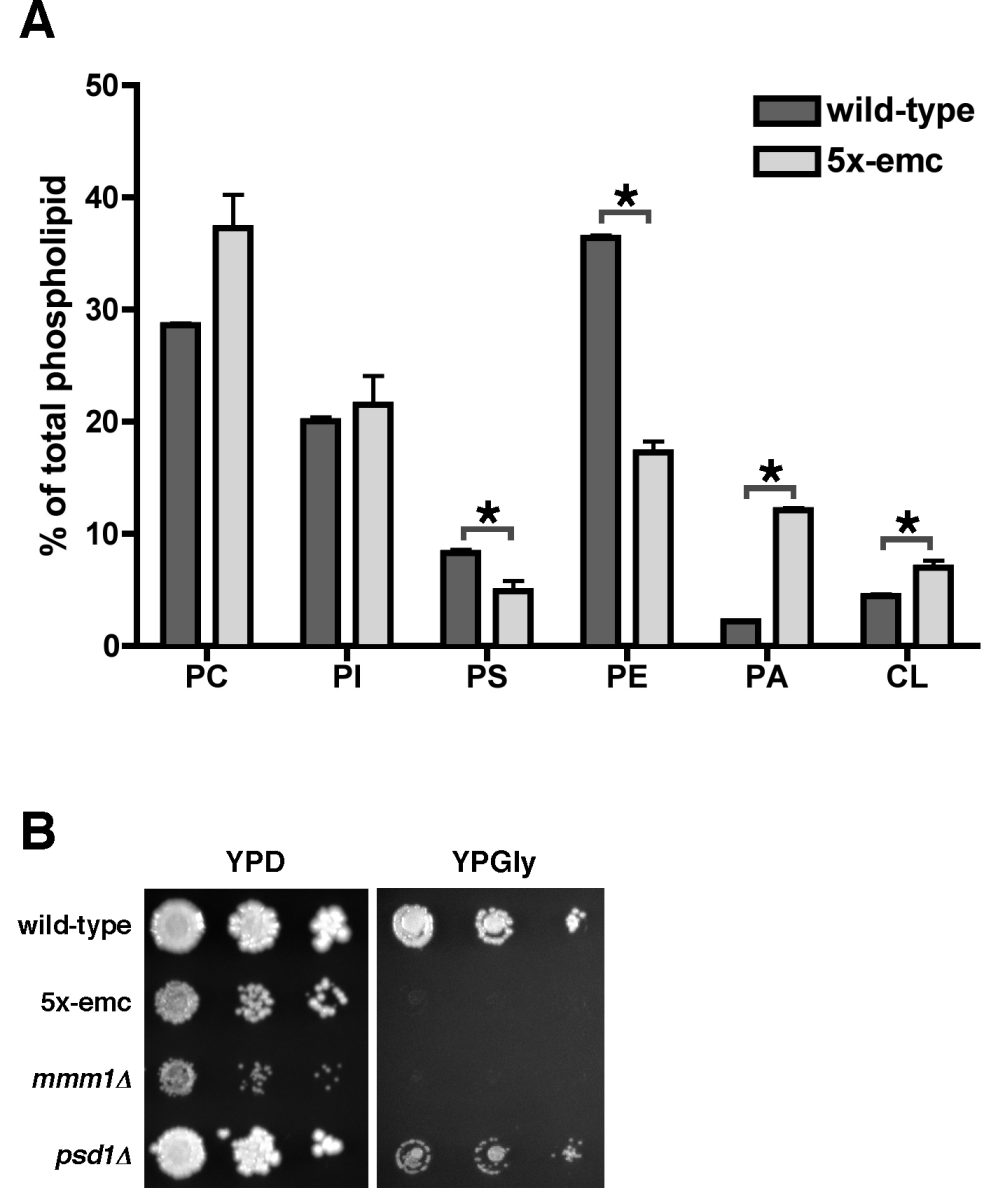
Mitochondria from cells missing Emc proteins have reduced levels of PS and PE and are not functional. (A) Wild-type and 5x-emc cells were grown for at least three generations in medium containing [^3^H]acetate, and the amount of the six major phospholipids in purified mitochondria was determined (mean ±s.d., *n* = 3 independent experiments). * *p*<0.05, independent two-tailed *t* test. The data used to generate this graph are in Table S5. (B) The 10-fold serial dilutions of the indicated strains on YPD and YPGly plates. The plates were incubated at 30°C for 3 d.

Because 5x-emc cells have reduced PS transfer from ER to mitochondria, we suspected that mitochondria from 5x-emc cells would have reduced amounts of PS and PE. To measure phospholipid levels, wild-type and 5x-emc cells were labeled with [^3^H]acetate for at least 3–4 generations and the relative abundance of the major phospholipids in purified mitochondria was determined. We found that PS levels in the mitochondria from 5x-emc cells were about 50% lower than those in wild-type mitochondria (Figure 5A). Therefore, reduced ER to mitochondria PS transfer in 5x-emc cells results in decreased steady-state PS levels in mitochondria. Notably, PE levels were also reduced about 50%. This reduction is probably caused by the defect in ER to mitochondria PS transfer in 5x-emc cells, as most PE in mitochondria is generated from PS by Psd1 [24]. Interestingly, the relative abundance of other phospholipids was increased in 5x-emc mitochondria, particularly phosphatidic acid (PA) and cardiolipin (CL) (Figure 5A). It may be that the transfer of PA and other CL precursors into mitochondria is not as sensitive to the loss of the EMC as is PS transfer. Alternatively, the increase in PA and CL may reflect a mechanism by which cells compensate for low levels of mitochondrial PE, which is thought to be critical for proper mitochondrial function [22].

Because the lipid profile of mitochondria in 5x-emc cells was dramatically altered, we wondered if the mitochondria were functional. Yeast strains with nonfunctional mitochondria cannot grow on media containing nonfermentable carbon sources such as glycerol. We found that 5x-emc cells and other strains missing multiple EMC proteins did not grow on the glycerol-containing medium, YPGly, as has previously been found for cells lacking the ERMES component Mmm1 (Figure 5B) [36]. Therefore cells missing multiple EMC proteins do not have functional mitochondria, probably because of the abnormal levels of phospholipids in the mitochondria of these strains. Interestingly, 5x-emc cells also had a substantial growth defect even on glucose-containing media (Figures 4B and 5B).

### 5x-emc Cells Have a Reduced Rate of ER to Mitochondria PS Transfer in Vitro

**Figure 6:**
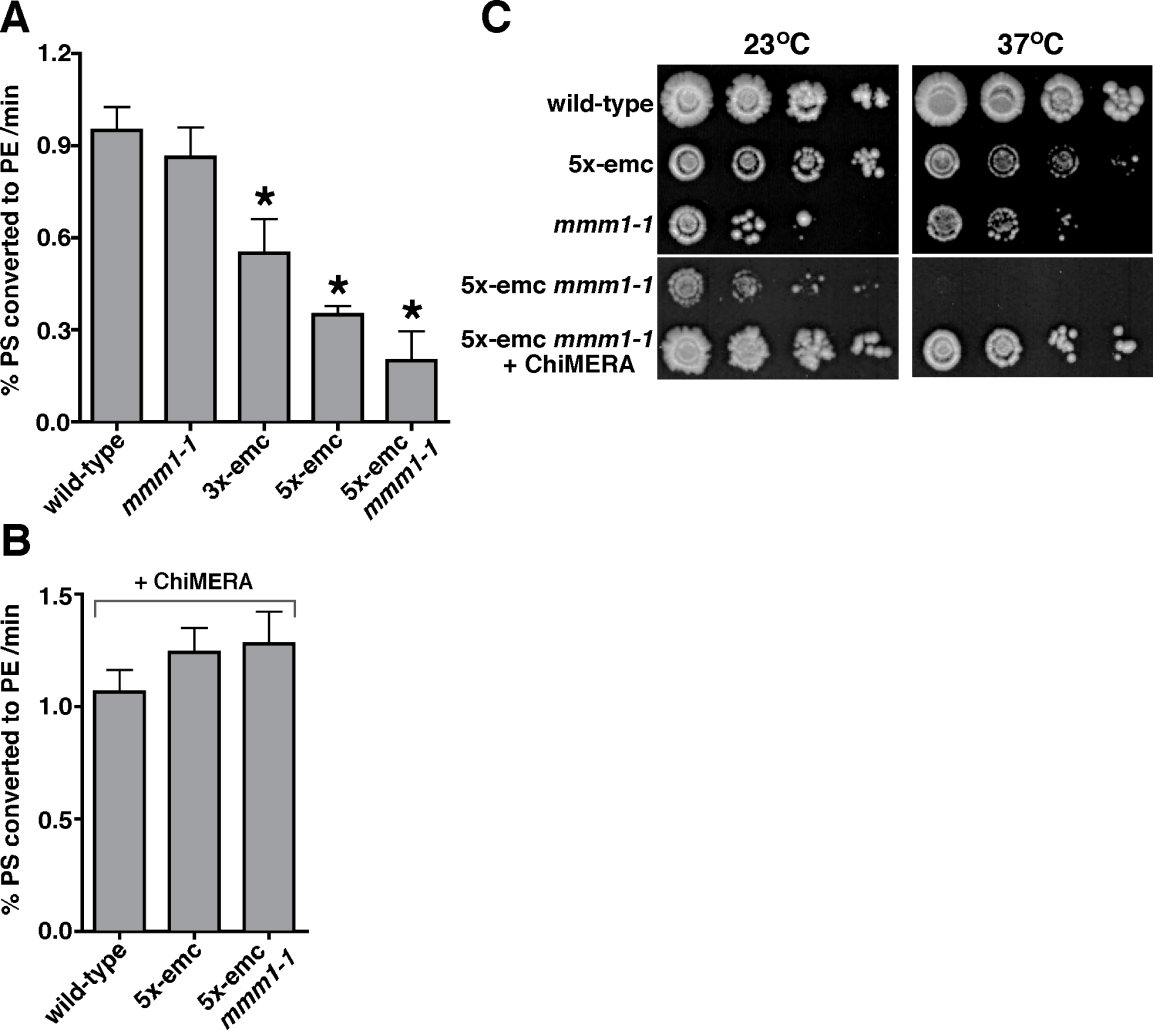
5x-emc *mmm1-1* cells are not viable and have a dramatic reduction in ER to mitochondria PS transfer at nonpermissive temperature. (A) Crude mitochondria were incubated with [^3^H]serine and Mn^2+^. After 20 min at 30°C, EDTA and an excess of unlabeled serine were added; chelation of Mn^2+^ by EDTA inhibits PS synthase and allows Psd1 to function. The samples were collected over 15 min, and the rate of [^3^H]PS to [^3^H]PE conversion per minute was calculated (mean ±s.d., *n* = 3–5 independent experiments). * *p*<0.05 compared to wild-type, two-tailed *t* test. (B) The rate of PS to PE conversion of strains expressing ChiMERA was determined as in (A) (mean ±s.d., *n* = 3 independent experiments). The data used to generate panels A and B are in Table S6. (C) Cultures of strains with the indicated genotypes were grown at 23°C and 10-fold serial dilutions were spotted on to YPD plates and incubated at 23°C or 37°C for 4 d.

Because we found that 5x-emc cells have reduced ER to mitochondria PS transfer in vivo, we wondered if a similar defect could be detected in vitro. We used a previously established two-step assay to monitor the transfer of newly synthesized PS from the ER to mitochondria [18],[37]. In the first step, crude mitochondria are incubated for 20 min with [^3^H]serine and Mn^2+^. We have shown that crude mitochondria have tightly associated ER-derived membranes that contain PS synthase [18]. The presence of Mn^2+^ is required by PS synthase but inhibits the conversion of [^3^H]PS to [^3^H]PE by Psd1 [37]. Thus, in the second step of the reaction, Mn^2+^ is chelated by EDTA and the PS to PE conversion rate was determined. Because Psd2 is not active in this assay [18], all PS to PE conversions in this assay are mediated by Psd1 and indicate the rate of PS transfer from ER to mitochondria. Using mitochondria derived from wild-type cells, we found that about 1% of [^3^H]PS synthesized was converted to PE per minute (Figure 6A). When mitochondria from 3x-emc (missing Emc2, Emc5, and Emc6) and 5x-emc cells were used, this rate decreased about 2- and 3-fold, respectively (Figure 6A). It should be noted that for all the strains tested, the rate of PS to PE conversion was linear (R^2^≥0.9). Because mitochondria derived from 5x-emc cells have Psd activity that is not lower than those from wild-type (Figure 4C), these findings indicate that the rate of ER to mitochondria PS transfer is significantly reduced in crude mitochondria derived from 5x-emc cells.

### The EMC and ERMES Complexes Are Required for Viability and ER to Mitochondria PS Transfer

Because the ERMES complex is thought to function as a tether between the ER and mitochondria, we wondered whether PS transfer would be slower in crude mitochondria missing Emc proteins and the ERMES complex. As disruption of a single ERMES component causes disassembly of the entire complex [14], we sought to delete one of the four genes encoding the ERMES proteins in 5x-emc cells. However, we were unable to delete *MMM1* in 5x-emc cells, probably because the resulting strain was not viable. Therefore, we introduced the conditional *mmm1-1* allele into 5x-emc cells. The 5x-emc *mmm1-1* strain was not viable at the nonpermissive temperature of 37°C (Figure 6B), indicating that cells required either ERMES or the EMC for viability.

We next asked if ER to mitochondria PS transfer was more reduced in cells missing both ERMES and the EMC than in cells missing only the EMC. We found that when 5x-emc *mmm1-1* cells were shifted to the restrictive temperature (37°C) they stopped growing after ∼4 h. Therefore, we isolated crude mitochondria from 5x-emc *mmm1-1* cells 3 h after shift to 37°C, to insure that the cells were still viable. In these mitochondria, the rate of PS to PE conversion was reduced ∼5-fold (Figure 6A). Because mitochondria derived from 5x-emc *mmm1-1* cells do not have less Psd activity than those from wild-type (Figure 4C), these findings suggested that cells missing both ERMES and the EMC have very little PS transfer from the ER to mitochondria, which was consistent with the growth defect of these cells. The difference between the rates for 5x-emc and 5x-emc *mmm1-1* mutants was not statistically significant, likely because of their already low rates of transfer. However, we found that the rate of PS to PE conversion in membranes derived from *mmm1-1* single mutant cells was not significantly lower than wild-type, suggesting that the EMC was the critical component mediating ER to mitochondria PS transfer in vitro.

### Cells Missing the EMC or the ERMES Complex Have Reduced ER–Mitochondrial Tethering

The decrease in ER to mitochondria PS transfer in cells missing EMC proteins could be caused by a decreased ability to transfer PS or by inefficient tethering of the ER to mitochondria. The EMC might play a direct role in these processes or it could regulate the proteins that mediate lipid transfer or tethering. To characterize the nature of the PS transfer defect in cells missing EMC proteins, we determined if artificially tethering the ER and mitochondria corrects the PS transfer defect in mitochondria derived from 5x-emc and 5x-emc *mmm1-1* cells. For these studies we used a fusion protein called ChiMERA, which has previously been shown to tether the ER and mitochondria [14]. When this fusion protein was expressed in 5x-emc and 5x-emc *mmm1-1* cells, it corrected the ER to mitochondria PS transfer defect in mitochondria derived from these strains (Figure 6B). It also restored the ability of 5x-emc *mmm1-1* cells to grow at elevated temperature (Figure 6C). These findings suggest that the defect in ER to mitochondria PS transfer in 5x-emc and 5x-emc *mmm1-1* cells may be caused by inefficient tethering of the ER and mitochondria.

**Figure 7:**
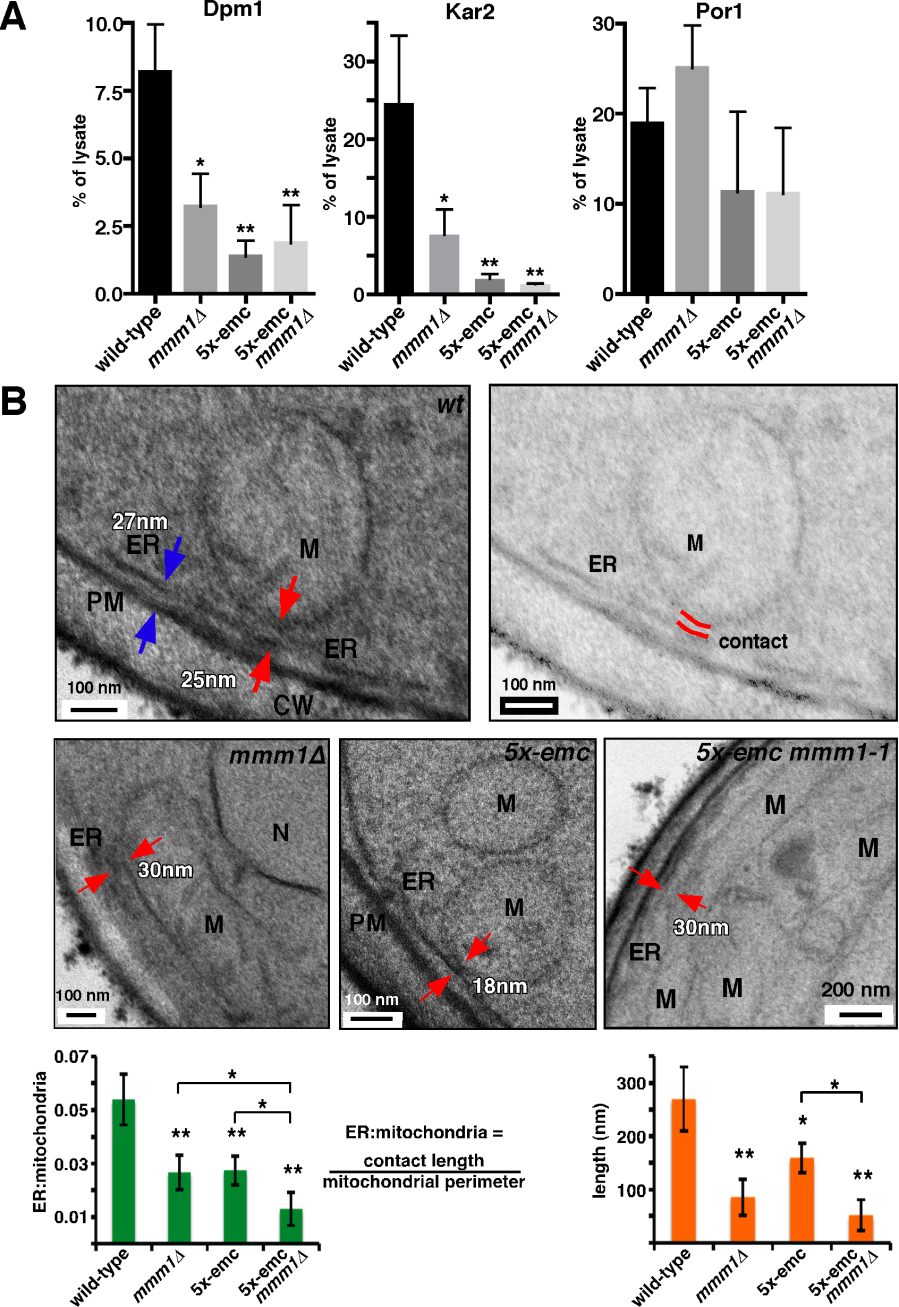
Cells lacking the EMC or the ERMES complex have reduced ER-mitochondria contacts. (A) Mitochondria were purified from the indicated strains and the percent of the ER proteins Dpm1 or Kar2 and the mitochondrial protein Por1 determined (mean ±s.d., *n* = 3 independent experiments). (B) Representative EM image shows a portion of a wild-type cell (top left). The same image is shown with reduced contrast (top right) to indicate the length of ER to mitochondria contact, highlighted with red lines. EM images showing portions of mutant cells are shown in the middle panels. Red arrows indicate a contact between the ER and mitochondria (M), and the blue arrows show a contact between the ER and the plasma membrane (PM). The bottom panels show the average ER–mitochondria ratio and average length of contacts (mean ±s.d., *n* = 15 individual cells). The ER–mitochondria ratio was determined by measuring the length of contacts between the ER and mitochondria divided by the length of mitochondrial perimeter in each image. * *p*<0.05, ** *p*<0.005 compared to wild-type, independent two-tailed *t* test. The data used to generate the graphs are in Table S7.

To more directly determine if cells missing the EMC or ERMES complexes have defects in ER–mitochondria tethering, we used a previously described assay to quantitatively measure the association of these organelles [12]. A subdomain of the ER, often called the mitochondrial-associated membrane (MAM), remains tightly associated with mitochondria after their purification, and is thought to be the portion of the ER that forms ER–mitochondria contacts. To quantitatively measure these contacts, we determined the percent of the ER that copurifies with mitochondria by measuring the fraction of the ER proteins, Dpm1 and Kar2, in purified mitochondria. We found a significant decrease in the percent for ER co-purifying with mitochondria from *mmm1Δ* or 5x-emc cells (Figure 7A). Because there were no significant differences in the percentages of mitochondria purified from the strains (Figure 7A, right panel), these results indicate that there is a dramatic decrease in ER–mitochondria tethering in *mmm1Δ* and 5x-emc cells.

We confirmed the decrease in ER–mitochondrial tethering in *mmm1Δ* and 5x-emc by ultrastructural analysis using transmission electron microscopy (EM). We quantified the length of contacts where the ER membrane was within a distance of 30 nm to the mitochondria membrane and the ratio of the length of ER–mitochondria contacts to mitochondrial perimeter. Contacts were significantly reduced in both *mmm1Δ* and 5x-emc cells (Figure 7B). There was also a significant difference between 5x-emc cells and 5x-emc *mmm1-1* cells, suggesting that both EMC and ERMES tether independently of one another. Notably, this is the first demonstration of a reduction in ER–mitochondrial tethering in cells missing the ERMES complex.

**Figure 8:**
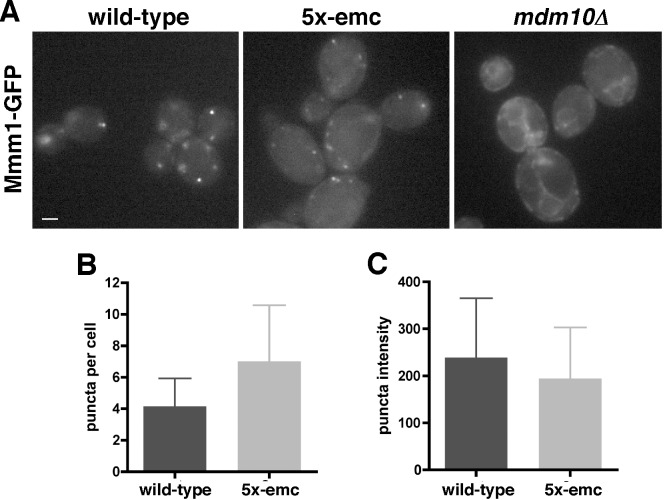
Formation of ERMES complex is not altered in 5x-emc cells. (A) Images of wild-type, 5x-emc, or *mdm10D* cells expressing Mmm1–GFP on the chromosome. Scale bar, 1 µm. (B) Number of puncta per cell in the indicated strains (mean ±s.d., *n* = 80 cells). (C) Average intensity of puncta in the indicated strains (mean ±s.d., *n* = 80 cells). * *p*<0.05 compared to wild-type, independent two-tailed *t* test. The data used to generate panels B and C are in Table S8.

The defect in ER–mitochondria tethering we found in 5x-emc cells was not because ERMES complex formation was compromised in these cells. In wild-type cells, the ERMES complex forms about 1–10 puncta per cell [14]. These structures are thought to form at sites of ER–mitochondria tethering. In cells missing any one of the four ERMES proteins, the remaining proteins do not form puncta [14]. Therefore, if ERMES puncta are visible in 5x-emc cells, the EMC is not necessary for ERMES assembly. Indeed, we found that the ERMES protein Mmm1-GFP forms puncta in 5x-emc cells (Figure 8A). In contrast, Mmm1-GFP was seen all over the ER in cells missing the ERMES protein Mdm10 (Figure 8A, right panel). We also compared the number and intensity of Mmm1-GFP puncta between wild-type and 5x-emc cells. The ERMES puncta in the mutant were more numerous but slightly less intense than those in the wild-type cells, indicating that ERMES complex formation is not dramatically affected in the mutant. Thus, the ERMES complex can still assemble in 5x-emc cells, indicating that the ER–mitochondria tethering defect in 5x-emc cells is likely independent of ERMES-mediated tethering. This finding suggests that there must be ER–mitochondrial tethering proteins in addition to the ERMES complex.

Even though the EMC is not required for ERMES assembly, it might play a role in the assembly of other tethering complexes, as it has been implicated in protein folding or quality control in the ER [29]–[33]. If there was a general defect in protein folding in mutants missing the EMC, the UPR might be induced in these cells. However, we found that the UPR was not induced in 5x-emc cells and that a UPR-responsive promoter is activated by ER stress in these cells (Figure S4).

### The EMC Interacts with Tom5 at ER–Mitochondria Contact Sites

**Figure 9:**
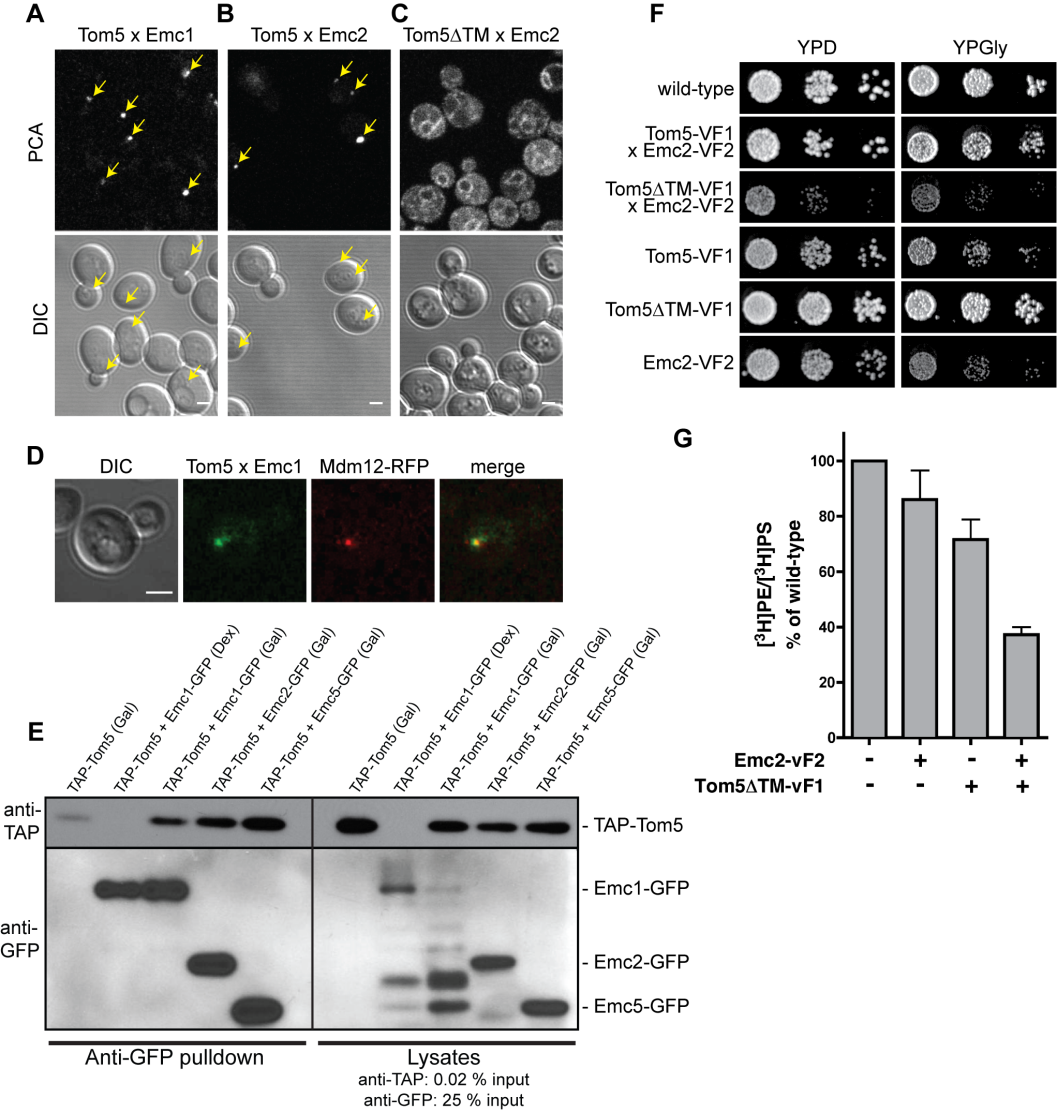
The EMC interacts with Tom5 at ER-mitochondria contacts. (A and B) Interactions between Tom5 and Emc1 (A) and Emc2 (B) proteins imaged by Venus PCA. (C) Interaction between Tom5ΔTM and Emc2 by PCA. (D) Colocalization of the Tom5–Emc interaction and Mdm12–RFP of the ERMES complex. (E) Coimmunoprecipitation of TAP–Tom5 and Emc1p, Emc2p, or Emc5p fused to GFP. Expression of TAP–Tom5 was induced in medium containing galactose (Gal) and repressed in medium with glucose (Dex). (F) Yeast growth assays for the indicated strains on media containing glucose (YPD) or glycerol (YPGly). Tom5 × Emc2 and Tom5ΔTM × Emc2 indicate haploid strains used for PCA containing Tom5 tagged with VF1 and Emc2 tagged with VF2. (G) Cells with the indicated genotypes were labeled with [^3^H]serine as in Figure 4A. The ratio of [^3^H]PS converted to [^3^H]PE was determined and expressed as a percent of wild-type cells (mean ±s.d., *n* = 3 independent experiments). * *p*<0.05 compared to wild-type, independent two-tailed *t* test. All scale bars, 2 µm. The data used to generate this graph are in Table S9.

Because the EMC might directly participate in ER–mitochondria tethering, we sought to identify proteins in the outer mitochondrial membrane with which it could partner. We searched for genes identified in the *CHO2* SGA screen that encoded mitochondrial outer membrane proteins. One gene, *TOM5*, showed a strong genetic interaction with *CHO2* that was rescued by choline (Table S1). *TOM5* encodes one of three small integral membrane proteins in the outer membrane of mitochondria, which are nonessential subunits of the translocase of the outer membrane (TOM) complex that imports proteins into mitochondria [38]. Thus, Tom5 was a good candidate for interacting with the EMC, and we used PCA to determine if it interacts with EMC proteins. We found that all the Emc proteins interacted with Tom5 in puncta (Figures 9A,B and S5A) that were suggestive of the localization of the ERMES complex [14]. Interestingly, when we deleted the transmembrane domain of Tom5, Tom5ΔTM now interacted with Emc2 on the ER (Figure 9C), indicating that insertion of Tom5 in the outer mitochondrial membrane was not required for its interaction with the EMC.

We next determined if the Emc–Tom5 PCA occurs at the same ER–mitochondria junctions where ERMES is localized. Remarkably, we found that in 100% of the cells we examined, the Emc1–Tom5 PCA puncta colocalized with ERMES foci (Figure 9D). This indicated that the EMC–Tom5 interaction likely formed a tether between ER and mitochondria at the same or nearby contact sites as defined by ERMES. We did not detect an interaction between Tom5 and another integral ER protein, Ale1, using PCA, even though Ale1–GFP localized throughout the ER similar to EMC proteins (Figure S5B). Ale1 has a cytoplasmically oriented C-terminus, which should be accessible to Tom5 on mitochondria [39]. These findings support a specific role for the EMC at contacts between ER and mitochondria.

To obtain more direct evidence for the interaction between the EMC and Tom5, we used co-immuoprecipitation. Lysates from cells expressing GFP-tagged Emc1, Emc2, or Emc5 and Tom5 fused to the tandem affinity purification (TAP) tag were immunoprecipitated with agarose beads conjugated to anti-GFP antibodies. TAP–Tom5 co-immunoprecipitated with all three of the Emc proteins we tested (Figure 9E). These results suggest that Emc proteins bind Tom5 and that the EMC may directly participate in ER–mitochondria tethering by interacting with the Tom complex. These data are also consistent with our PCA data showing that all the Emc proteins interact with Tom5, which may explain why it was necessary to ablate a number of these proteins to obtain significant defects in PS transfer from the ER to mitochondria.

Next we examined the functional relevance of the EMC–Tom5 interaction. To begin, we deleted *TOM5* and measured PS transfer using the in vivo PS transfer assay; we found no PS transfer defect in cells missing Tom5. However, we noticed that the PCA interaction on the ER between Emc2 and Tom5ΔTM caused cells to grow very poorly (Figure 9F), whereas the PCA with full-length Tom5 grew similarly to wild-type, suggesting that the interaction of nonmitochondrial Tom5ΔTM with the EMC on the ER interfered with tethering and PS transfer from the ER to mitochondria. Therefore, we measured PS transfer in vivo in cells expressing either Emc2–VF2 or Tom5ΔTM–VF1 or both (VF1 and VF2 are the two halves of the Venus fluorescent protein used in the PCA). ER to mitochondria PS transfer was decreased to ∼40% of that of wild-type in cells expressing both Emc2–VF2 and Tom5ΔTM–VF1, whereas transfer was only modestly decreased in cells expressing only Tom5ΔTM–VF1 (Figure 9G). Therefore, the PCA between Tom5ΔTM and Emc2 slowed PS transfer from the ER to mitochondria, suggesting the Emc may directly tether the ER and mitochondria by interacting with Tom5 in the outer mitochondrial membrane, and that this interaction is disrupted by the PCA between Emc2 and Tom5ΔTM.

## Discussion

Lipid exchange between the ER and mitochondria is critical for mitochondrial membrane biogenesis and lipid metabolism. We found that cells missing multiple components of a conserved protein complex in the ER, called EMC, had dramatic reductions in the amount of PS transferred from the ER to mitochondria both in vitro and in intact cells. These cells also had significantly lower levels of PS and PE in mitochondria. We found that the reduced ER to mitochondria PS transfer is probably caused by a significant reduction in the extent of tethering between the ER and mitochondria. Finally, we also demonstrate a role for the EMC in phospholipid metabolism and mitochondrial function by our unbiased global genetic analyses. Taken together, these findings strongly support that the EMC mediates lipid trafficking between the ER and mitochondria.

We propose the EMC mediates ER–mitochondrial tethering independent of the previously identified ERMES complex. A number of findings support this claim. First, we found a significant decrease in the amount of ER associated with mitochondria both by determining the fraction of ER that copurified with mitochondria and by EM. Second, the PS transfer defect in cells missing multiple EMC proteins was ameliorated by expression of the artificial ER–mitochondria tether, ChiMERA. Third, we found a strong aggravating genetic interaction between the 5x-emc mutant and an ERMES temperature-sensitive allele, *mmm1-1*, suggesting that the EMC contributes to an essential overlapping function with the ERMES. And finally, expression of ChiMERA completely rescued the synthetic lethality of the 5x-emc *mmm1-1* combined mutant, confirming that this lethality arose as a result of deficient ER–mitochondrial tethering.

How might the EMC mediate ER–mitochondrial tethering? Because the EMC has been implicated in protein folding and quality control in the ER [29]–[33], the EMC could be necessary for the folding or processing of proteins directly involved in tethering (or lipid transfer), rather than participating in tethering directly. However, we found that ERMES complex assembly was not compromised in 5x-emc cells, indicating that the tethering defect we found in this strain was not due to a failure of ERMES proteins to assemble. We also found that in 5x-emc cells the UPR was not induced and occurred normally in response to ER stress (Figure S4). Importantly, we provide evidence by two independent methods that multiple Emc proteins interact with the mitochondrial outer membrane protein Tom5, suggesting that the EMC–Tom5 interaction is important for tethering ER and mitochondria. Consistent with this, loss of the mitochondrial anchor for this tether by deleting the C-terminal transmembrane domain of Tom5 interfered with PS transfer to mitochondria and disrupted mitochondrial function. Therefore, we favor a model in which the EMC directly facilitates ER–mitochondrial tethering.

Our findings indicate that the EMC and ERMES perform tethering functions independently of one another, as we found a significant decrease in tethering in mutants missing either complex. Whether the tethering defects due to loss of either complex are additive remains unclear. Why there are multiple tethering complexes is not yet known, but a plausible explanation is that each tether may facilitate a different type of communication between the ER and mitochondria. Given that there is a significant decrease in ER to mitochondria PS transfer in cells missing EMC, but not in cells lacking ERMES [17],[18], it may be that ERMES facilitates the exchange of other lipids or perhaps is involved in other forms of signaling/transport between the ER and mitochondria. Another possibility is that the proteins necessary for PS transfer associate with the EMC but not ERMES (Figure S6). Another notable difference between the EMC and ERMES is that only the EMC is conserved in higher eukaryotes [29],[40]. Whether the mammalian EMC homologue also facilitates ER–mitochondria tethering and PS transfer remains an important question.

Interestingly, our findings also suggest that 5x-emc cells have lipid metabolism defects in addition to reduced PS transfer between the ER and mitochondria. We found that 5x-emc *psd2Δ* cells were not viable and did not grow even when supplemented with ethanolamine. Because *psd1*Δ *psd2*Δ cells grow when ethanolamine is present in the medium, 5x-emc *psd2Δ* cells must have greater defects in lipid metabolism in addition to altered PS transfer to Psd1 in mitochondria. This conclusion is further supported by the results of our SGA screen with *EMC6*, which identifies functional links to global transcriptional control of phospholipid metabolism (*INO2*, *INO4*) and fatty acid biosynthesis (*HTD2*, *MCT1*). It is also likely that the EMC has functions in addition to its role in ER-mitochondrial membrane tethering, as indicated by other studies and as is implied by its uniform localization throughout the ER, which is not restricted to contacts [29]–[33].

One of the more perplexing aspects of EMC function is the apparent redundancy between individual Emc proteins and Tom5 in tethering and PS transfer. That is, why is deletion of individual Emc proteins or Tom5 not sufficient to reduce PS transfer to mitochondria? Although there is no shared sequence identity amongst Emc proteins that would suggest obvious functional redundancy, our finding that multiple Emc proteins interact with Tom5 provides a sound alternative explanation for functional overlap in tethering. However, one would then expect that deletion of Tom5 would disrupt tethering and PS transfer, similarly to loss of multiple Emc proteins. That this appears not to be the case suggests that there is additional redundancy in tethering on the mitochondrial membrane, perhaps involving other TOM complex proteins. Although there is no sequence identity between Tom5 and the two other small TOM subunits, Tom6 and Tom7, all these proteins are similar in size and topology and appear to be functionally redundant within the TOM complex [41],[42]. Hence, the EMC may interact with Tom6 and Tom7, and, indeed, preliminary PCA evidence indicates that Tom6 and Tom7 interact with certain Emc proteins (unpublished data). A role for the TOM complex in tethering between the mitochondrial outer and inner membranes through interaction with the translocase of the inner membrane (TIM) complex is well established [43]. Thus, our work further implies that tethering between TOM and the EMC, and TOM and TIM may facilitate efficient PS transfer from the ER to the inner mitochondrial membrane, the location of PS decarboxylation by Psd1 [16].

## Materials and Methods

### Strains, Plasmids, and Growth Media

Strains and plasmids used in this study are listed in Table S13. Media used were YPD (1% yeast extract, 2% peptone, 2% glucose), YPGly (1% yeast extract, 2% peptone, 3% glycerol), and synthetic complete (SC) media (2% glucose, 0.67% yeast nitrogen base without amino acids, and amino acid dropout mix from BIO101). Where indicated, ethanolamine or choline was added to a concentration of 5 mM. 5-FOA was added at a concentration of 1 mg/ml.

Single deletion strains were obtained from freezer stocks of the haploid yeast deletion collection (BY4741, Mat a, KanMX, a gift from C. Boone) unless otherwise stated. Other gene deletions were constructed using the PCR method with the heterologous markers S. pombe *HIS5* (pKT128), K. lactis *URA3* (pKT209), or *NatR* (p4339). Double deletion strains were derived from the meiotic products of heterozygous diploids with at least three spores of each genotype being compared. All yeast cells expressing GFP fusion proteins were tagged endogenously in haploids unless otherwise indicated. C-terminally tagged GFP strains were constructed by standard methods involving single-step gene replacement using the pKT128 (SpHIS5) plasmid [44] in the wild-type Y7043 background and crossed to the indicated single deletion mutants (BY4741; kanMX). Mdm12–RFP was created similarly to C-terminal GFP fusions except using pMRFP–NAT in BY4741. For PCA, the plasmids used for C-terminal genomic tagging were created as follows: Venus–YFP fragments F1 and F2 were amplified by PCR from p413–TEF–Zip linker–Venus YFP-F1 and p415–TEF–Zip linker–Venus YFP-F2 (gift of S. Michnick [45]), and cloned into plasmids pKT128 and pKT209 [44], respectively, replacing yEGFP and including a myc tag (MEQKLISEEDL) in the linker region to give pHVF1CT (pFA6a–myc–VF1–HIS5) and pUVF2CT (pFA6a–myc–VF2–URA3). For N-terminal integrations, plasmid pHVF1NT (HIS5–PHO5–VF1) was created by replacing eGFP in plasmid pTLHPG (HIS5–PHO5–GFP; gift of T. Levine) with the Venus F1 PCR fragment. To create the Tom5ΔTM–VF1 strain, the C-terminal 21 amino acids of endogenous *TOM5* were replaced in frame by VF1.

### SGA Analysis for CHO2 and EMC6

SGA analysis was performed according to established protocols [46] essentially as previously described [47] using a Singer RoToR Colony Arraying robot (Singer Instruments). *Δcho2*::URA3 and *Δemc6*::URA3 query strains were constructed using standard techniques in strain background Y7092 and crossed to the yeast haploid deletion mutant array (DMA) using a Singer RoToR HDA robot. Following diploid selection, spots were replicated three times and sporulated for 5 d. Haploids were germinated on SD-media lacking histidine, arginine, and lysine supplemented with thialysine and canavanine (both at 100 µg/ml). Control sets of single deletion strains were generated by plating on media containing 5-FOA to counterselect for the *Δcho2*::URA3 or *Δemc6*::URA3 alleles and G418 sulfate (200 µg/ml) to select for the DMA strain, whereas double mutants were selected for by plating on media lacking uracil and containing G418 sulfate. A further round of selection was performed on the same media. For the *CHO2* SGA screen in the presence of choline, all plates additionally contained 1 mM choline. Arrays were imaged using a flatbed scanner. Balony software (http://code.google.com/p/balony/) was used to measure spot sizes, determine cutoff values for genetic interactions, and define strains that showed statistically significant changes in growth rate [48]. Cutoff values for genetic interactions were defined for each screen by determining three standard deviations from the mean of the ratios of the double mutant to single mutant growth rates. Double mutant strains that met the cutoff and showed significant changes in growth relative to the corresponding single mutant control (one-tailed student's *t* test; *p*<0.05; *n* = 3) were considered as genetic interactions. For the *CHO2* SGA screen, aggravating genetic interactions identified in the screen done in the absence of choline were considered rescued if they were no longer identified as genetic interactions according to the above criteria in the screen done in the presence of choline. Gene ontology analysis was performed using Funspec (http://funspec.med.utoronto.ca) and Cytoscape (http://www.cytoscape.org) [49],[50].

### Fluorescence Microscopy

Log phase live yeast cells were imaged using a Zeiss LSM-5 Pascal confocal microscope and Zeiss Pascal software. Unless otherwise stated, all proteins were tagged at the C-terminus of the endogenous protein. Optical slices were taken through the center of each cell, and images being directly compared were captured with identical microscope settings on the same day.

For the studies with cells expressing Mmm1–GFP, cells were imaged live at room temperature by using an Olympus BX61 microscope, a UPlanApo ×100/1.35 lens, a QImaging Retiga EX camera, and IVision software (version v 4.0.5).

### PCA

The Venus–YFP variant of PCA was used to examine protein–protein interactions in live yeast. Unless otherwise stated, endogenous proteins were tagged in haploid yeast by the PCR method with either VF1 or VF2 in the BY4741 and Y7043 strains, respectively. Correct integration and expression was confirmed by colony PCR and Western blot analysis with anti-myc antibodies (Sigma) for each fusion protein. The VF1 and VF2 strains to be assayed were then crossed, and haploid meiotic progenies with both alleles were recovered by random spore analysis or tetrad dissection. Finally, the PCA was visualized in log phase yeast by confocal microscopy.

### In Vivo Labeling with [^3^H]serine

Cells were labeled with L-[3-^3^H]serine (American Radiolabeled Chemicals) as described [35] with the following modifications. About 2 OD_600_ units of cells from a saturated culture were added to 25 ml of SC medium and incubated at 30°C. When the cultures reached an OD_600_ of about 0.3, 10 µg/ml myriocin (SigmaAldrich, stock  = 500 µg/ml in methanol) was added to the medium, the cells were grown for 30 min, and 10 µg/ml cerulenin (SigmaAldrich, stock  = 5 mg/ml in dimethyl sulfoxide) was added to the medium. About 5 min later, 50 µCi of [^3^H]serine was added to the medium, and the cells were grown for an additional 30 min. The culture was then added to an equal volume of ice-cold water, and it was washed once with ice-cold water. Cells were lysed in a Mini-BeadBeater-8 (BioSpec). Lipids were extracted as described [51], separated by HPLC [52], and the fractions containing PS, PE, and PC were collected and analyzed by liquid scintillation counting.

### Mitochondrial Extracts and in Vitro [^3^H]serine Labeling

Crude mitochondria were prepared as described [18]. Briefly cells were grown in YPD medium to an OD_600_ of ∼0.3, washed once with water, and incubated in 1 ml 0.1 M Tris-SO_4_ (pH 9.4) containing 10 mM DTT for 10 min at 30°C. They were washed once with spheroplast buffer (1.2 M sorbitol, 20 mM Tris pH 7.4) and resuspended in 1.5 ml of the same buffer containing 1 mg/ml zymolyase 20T (Seikagaku Biobusiness, Japan). After incubation for 60 min at 30°C, cells were pelleted (5 min, 500×*g*) and washed twice with spheroplast buffer. Cells were resuspended in ice-cold lysis buffer (0.6 M mannitol, 2.0 mM Tris pH 7.4, 1 mM EDTA, 1 mM PMSF and protease inhibitors, [Roche]) and lysed with a dounce using a B-pestle. The extract was centrifuged twice for 5 min at 3,000×*g* to remove unlysed cells and debris. The supernatant was centrifuged at 9,600×*g* for 10 min and the pellet containing crude mitochondria was resuspended in lysis buffer using a dounce (B-pestle).

The method of labeling crude mitochondria with [^3^H]serine was adapted from [37]. We heated 1–2 mg of crude mitochondria in 1 ml of lysis buffer to 30°C, and 0.6 µa MnCl_2_ and 10 µCi of L-[3-^3^H]serine (American Radiolabeled Chemicals) were added. After 20 min, 0.5 mM serine and 5 mM EDTA were added. Samples of 200 µl were taken after 0, 5, 10, and 15 min and added to 6 ml of chloroform:methanol (1∶2). Lipids were extracted, separated by HPLC, and extracted as described in the previous section.

### Metabolic Labeling with [^3^H]ethanolamine

Forty OD_600_ of yeast cells were grown to mid-log phase in SC media and collected by centrifugation. After washing once in 0.67% w/v Yeast Nitrogen Base, cells were resuspended in 12 ml of 0.67% yeast nitrogen base containing 2% dextrose and 12 µCi [^3^H]ethanolamine (Perkin-Elmer). After labeling for 30 min at 37°C, the suspension was diluted to a final volume of 120 ml in synthetic defined media. The culture was placed in a shaking incubator at 30°C and 20 ml aliquots taken every 30 min. Lipids were extracted and separated by HPLC as described in the previous section.

### Mitochondrial Purification and Determination of Mitochondrial Lipid Content

About 2 OD_600_ units of cells from a saturated culture were washed with water, resuspended in 50 ml of fresh SC medium containing 200 µCi of [^3^H]acetate (American Radiolabeled Chemicals), and grown at 30°C for at least 3–4 generations. Crude mitochondria were purified as described in the previous section and further purified by equilibrium centrifugation using density gradients made from OptiPrep (Axis-Shield, Oslo, Norway) as described [53]. Lipids were extracted and separated by one-dimensional TLC as described by [54]. TLC plates were scanned on a RITA Star Thin Layer Analyzer (Raytest).

### Quantification of ER Co-Purified Along with Pure Mitochondria

Mitochondria were purified as described in the previous section. Portions of the cell lysates and purified mitochondria were immunoblotted using antibodies against the ER-membrane proteins Dpm1 (Invitrogen) or Kar2 (gift from R. Schekman) and the mitochondrial membrane protein Por1 (Invitrogen). The immunoblots were quantitated using the Odyssey infrared imaging system (LI-COR Biosciences), and the percentage of each marker in the purified mitochondria was determined.

### Psd Assay

Psd assays were performed as described [35] except that the concentration of the substrate, 1-oleoyl-2-[12-[(7-nitro-2-1,3-benzoxadiazol-4-yl)amino]dodecanoyl]-*sn*-glycero-3-phosphoserine (Avanti Polar Lipids), was 500 µM.

### Co-Immunoprecipitation of Emc Proteins and Tom5

Yeast cultures were grown in YP+2% galactose or YP+2% dextrose to express or suppress TAP–Tom5, respectively. Log phase cultures (30 OD_600_ units) were collected by centrifugation and resuspended in 400 µl of lysis buffer (250 mM NaCl, 50 mM Tris-HCl pH 7.4, 50 mM NaF, 5 mM EDTA, 1 mM DTT, 1 mM AEBSF, and 0.1% NP-40). Cells were disrupted with glass beads for 5 min at 4°C. The resulting lysate was centrifuged at 16,100×g for 5 min, and a sample of the supernatant was collected. The supernatant was then incubated under rotation with 20 µl of prewashed GFP–Trap_A bead slurry (Chromotek) for 1 h at 4°C. The supernatant was removed and the beads were washed with lysis buffer (3×1 ml, then 3×500 µl with 5-min incubations). Beads were resuspended in 100 µl of sample buffer (50 mM Tris-HCl pH 6.8, 10% glycerol, 2% SDS, 4% 2-mercaptoethanol, and 0.02% bromophenol blue) and eluted by heating at 65°C for 20 min. The eluted protein was subjected to SDS-PAGE (10% polyacrylamide) and immunoblotted with anti-TAP (1∶5,000 dilution, Pierce, CAB1001) or anti-GFP (1∶5,000 dilution, Roche, 11814460001) antibodies. Goat anti-rabbit IgG-HRP (1∶5,000 dilution, Bio-Rad, 172-1019) and goat anti-mouse IgG-HRP (1∶5,000 dilution, Bio-Rad, 172-1011) were used as secondary antibodies. Blots were imaged with either Supersignal West Pico or Femto ECL substrate (Thermo Scientific).

### EM

Cells were grown to mid-logarithmic growth phase, and 10 OD_600_ units of cells were fixed in 1 ml of fixative media (1% glutaraldehyde, 0.2% paraformaldehyde, and 40 mM potassium phosphate, pH 7.0) for 10 min at room temperature. The cells were pelleted and resuspended in 1 ml of fresh fixative media and incubated on ice for 50 min, washed twice with 0.9% NaCl and once with water. They were then incubated with 2% solution of KMnO_4_ for 5 min at room temperature, centrifuged, and resuspended with a freshly prepared solution of 2% KMmO_4_ for 45 min at room temperature for en-bloc staining. The cells were then dehydrated using a graded series of ethanol solutions (50%, 70%, 80%, 90%, 95%, and 100%) for 10 min each, with two more incubations in 100% ethanol from a freshly opened bottle. The samples were subsequently embedded stepwise using Spurr's low viscosity resin (EMS, Hatfield, PA). Samples were infiltrated for 2 h each with a 3∶1, 1∶1, and 1∶3 dehydrating agent–embedding media mixture. Cells were incubated overnight with 100% fresh resin. The next day, the cells were resuspended in fresh 100% resin for 2–3 h, transferred into BEEM embedding capsules, and polymerized at 70°C for 72 h. Semi- and ultrathin sections were produced with a diamond knife (Diatome, Biel, Switzerland) on an ultra-microtome (Ultracut UCT, Leica-Microsystems), collected on 200 mesh copper grids (EMS, Hatfield, PA), poststained with uranyl acetate and lead citrate, and visualized with a FEI Tecnai T12 TEM, operating at 120 kV. Pictures were recorded on a below mounted Gatan 2k×2k CCD camera.

Contact sites between the ER and mitochondria were defined as regions where these organelles come within 30 nm of one another. The ER–mitochondria ratio was determined by measuring the length of contacts between the ER and mitochondria divided by the length of mitochondrial perimeter in each image.

### β-Galactosidase Assay

Cells were transformed with pMCZ-Y, a plasmid encoding the lacZ gene under the *KAR2* promoter. Where indicated, 1 mM DTT was added to growing cells 1 h before the assay. β-Galactosidase assay was performed as described [55].

## Supporting Information

Figure S1 **Yeast growth assays of mutants identified in the** ***CHO2*** **SGA screen, related to** **Figure 1** **.** Serial dilutions of the indicated strains were spotted onto agar plates containing SC medium with or without ethanolamine or choline.
(TIF)

Figure S2 **PS synthesis and conversion to PE is linear in 5x-emc cells, related to** **Figure 4A** **.** (A) Total amount of [^3^H]PS synthesized in the experiments in Figure 4A (total [^3^H]PS synthesized  =  [^3^H]PS + [^3^H]PE); mean ±s.d., *n* = 2–5 independent experiments. (B) 5x-emc cells were labeled as in Figure 4A, and the amount of radiolabeled PS and PE per OD_600_ was determined (mean ±s.d., *n* = 3 independent experiments). The data used to generate these graphs are in Table S10.
(TIF)

Figure S3 **PE synthesis by the Kennedy pathway and PE methylation are not reduced in 5x-emc cells, related to** **Figure 4B** **.** Wild-type and 5x-emc cells were labeled with [^3^H]ethanolamine for the indicated times, and lipids were extracted, separated, and quantified by HPLC and scintillation counting (mean ±s.d., *n* = 3). Total counts in PE and PC were not significantly different between wild-type and 5x-emc at each time point. The ∼1.7-fold increase in PC synthesized in the 5x-emc mutant at 90 min was significant (*p*<0.005). The data used to generate these graphs are in Table S11.
(TIFF)

Figure S4 **The UPR is not induced in 5x-emc cells, related to** **Figure 8** **.** Wild-type and 5x-emc cells were transformed with pMCZ-Y, a high copy plasmid carrying the lacZ gene under the *KAR2* promoter. Cells were grown to mid-logarithmic growth phase in the presence or absence of 1 mM DTT for 1 h, and β-Galactosidase activity was determined (mean ±s.d., *n* = 3). (A) Mean β-Galactosidase activity. (B) Ratio (fold increase) of β-Galactosidase activity between cells with and without DTT. The data used to generate these graphs are in Table S12.
(TIF)

Figure S5 **Interaction of other Emc proteins and Tom, related to** **Figure 9A** **.** (A) PCA interaction of Tom5 and the indicated Emc proteins were visualized as in Figure 9A. (B) Ale1 control for PCA between the EMC and Tom5, related to Figure 7. Emc1 × Tom5 PCA (left panels) and Ale1 × Tom5 PCA (center panels) in diploids captured with identical microscope settings. Ale1 tagged at the endogenous gene locus with GFP (right panels). All scale bars, 2 µm.
(TIF)

Figure S6 **Model of the role of tethering in PS transfer from ER to mitochondria.** In wild-type cells (top left), tethers are formed by both ERMES (red) and the interaction of the EMC (blue) with the TOM complex (brown). A putative transport complex (purple) associates with the EMC and facilitates PS transfer from the ER to mitochondria. In cells missing ERMES (top right), tethering is reduced but the transporter can still function at contacts mediated by the EMC. In cells missing the EMC (bottom right), the transporter is not enriched at contact sites and PS transport is reduced. When ChiMERA (green) is expressed in cells missing the EMC (bottom right), the increased tethering of the ER and mitochondria allows the PS transporter to function.
(TIF)

Table S1 ***CHO2*** **aggravating genetic interactions rescued by choline, related to** **Figure 1** **.** Aggravating genetic interactions with *CHO2* and rescue by 1 mM choline determined using Balony software are listed. Genetic interaction strength is plotted as the ratio (Ratio) of the growth of the double mutant with *Δcho2* (Exp) versus the *Δcho2* single mutant (Ctrl) and as the difference (Diff) in growth by subtracting the growth of the single mutant (Ctrl) from the double mutant (Exp). The *p* values were calculated using a one-tailed Student's *t* test by comparing growth of the single versus double mutants (*n* = 3).
(XLS)

Table S2 **Functional group enrichment for genes identified in the** ***CHO2*** **SGA screen, related to** **Figure 1** **.** Only genes whose interactions were rescued by choline (Table S1) were used in the analysis.
(XLS)

Table S3 **Genetic interactions with** ***EMC6*** **, related to** **Figure 2** **.** Aggravating and alleviating genetic interactions identified by SGA analysis are listed along with the strength of the interactions, plotted as in Table S1.
(XLSX)

Table S4 **Data for graphs in** **Figure 4A and 4C** **.**
(XLS)

Table S5 **Data for graph in** **Figure 5A** **.**
(XLS)

Table S6 **Data for graphs in** **Figure 6A and 6B** **.**
(XLS)

Table S7 **Data for graphs in** **Figure 7A and 7B** **.**
(XLS)

Table S8 **Data for graphs in** **Figure 8B and 8C** **.**
(XLSX)

Table S9 **Data for graphs in** **Figure 9G** **.**
(XLS)

Table S10 **Data for graphs in Figure S2A and S2B.**
(XLS)

Table S11 **Data for graphs in Figure S3.**
(XLS)

Table S12 **Data for graphs in Figure S4A and S4B.**
(XLS)

Table S13 **Strains and plasmids used in this study.**
(XLS)

## Abbreviations

5-FOA: 5-fluoroorotic acid
CL: cardiolipin
DAG: diacylglycerol
EM: electron microscopy
EMC: endoplasmic reticulum membrane protein complex
ER: endoplasmic reticulum
ERMES: ER–mitochondria encounter structure
MAM: mitochondrial-associated membranes
PA: phosphatidic acid
PC: phosphatidylcholine
PCA: protein-fragment complementation assay
PE: phosphatidylethanolamine
PS: phosphatidylserine
SGA: synthetic genetic array
TAP: tandem affinity purification
TOM: mitochondrial translocase of the outer membrane
UPR: unfolded protein response
