# Supplementary figures and images for: A Conserved Endoplasmic Reticulum Membrane Protein Complex (EMC) Facilitates Phospholipid Transfer from the ER to Mitochondria
Source: PLoS Biol. 2014 Oct 14;12(10):e1001969. doi: 10.1371/journal.pbio.1001969 (PMC4196738; doi:10.1371/journal.pbio.1001969)

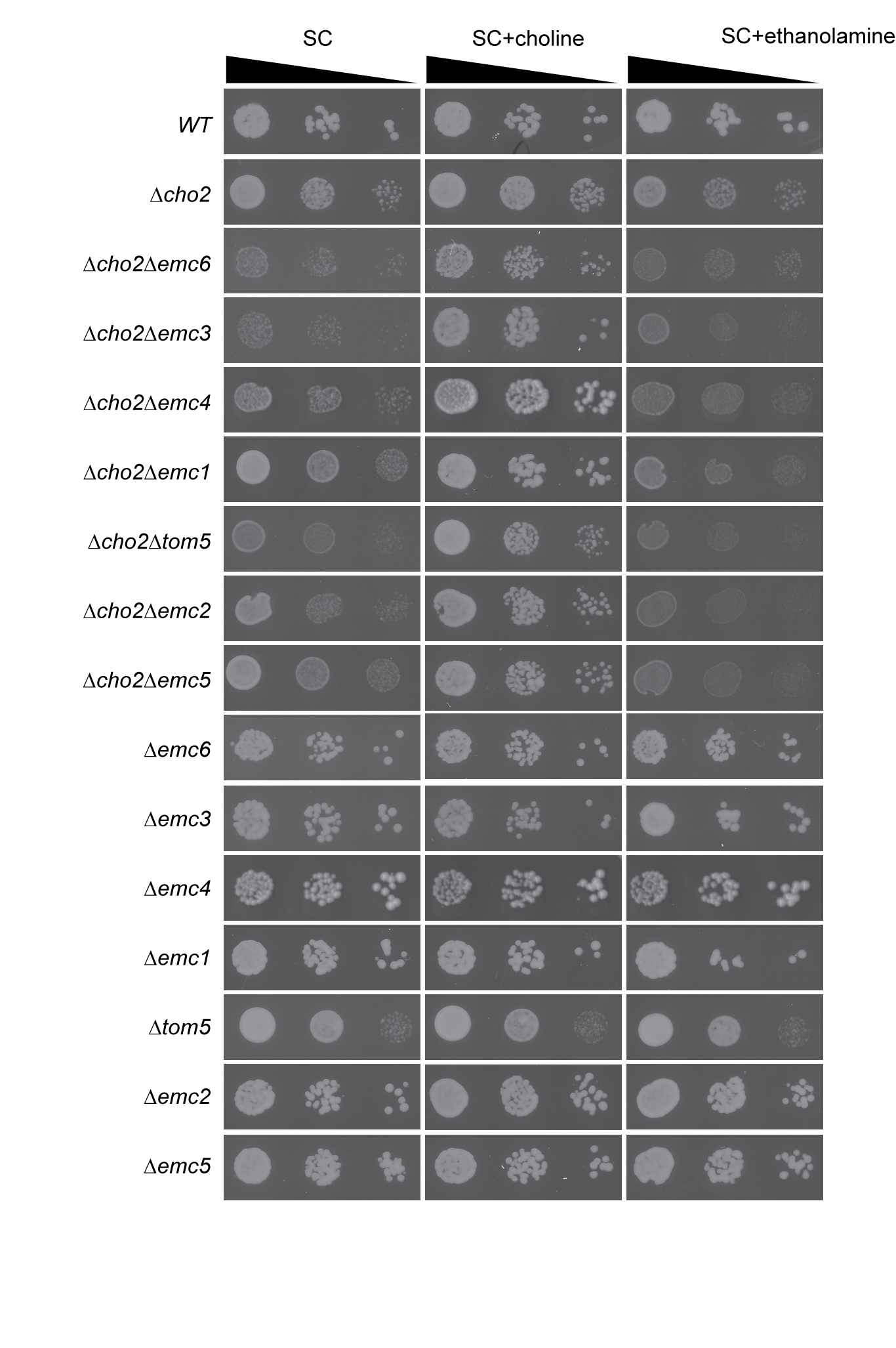

Supplement: Figure S1 — Yeast growth assays of mutants identified in the CHO2 SGA screen, related to Figure 1 . Serial dilutions of the indicated strains were spotted onto agar plates containing SC medium with or without ethanolamine or choline. (TIF) [file pbio.1001969.s001.tif]

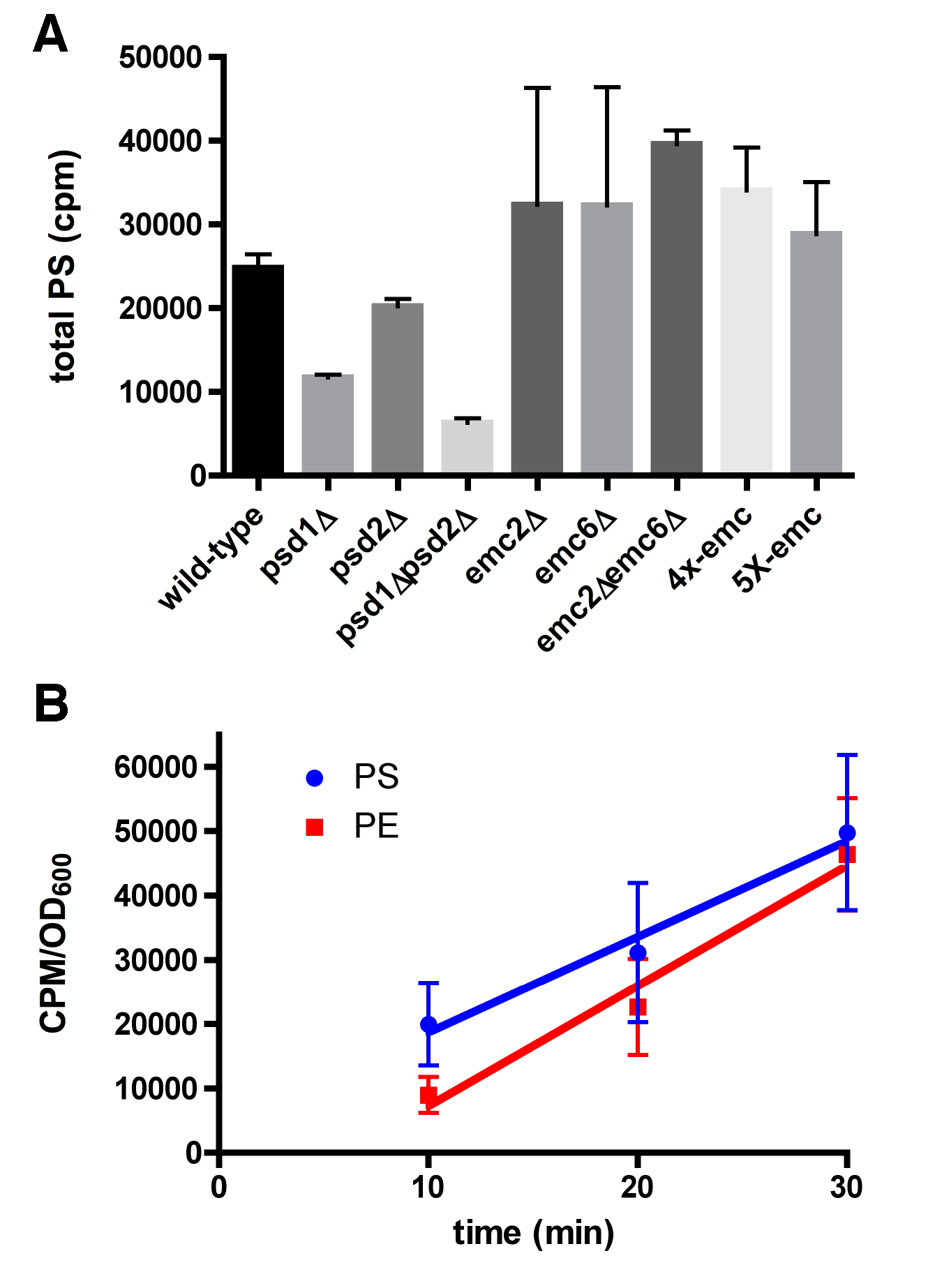

Supplement: Figure S2 — PS synthesis and conversion to PE is linear in 5x-emc cells, related to Figure 4A . (A) Total amount of [3H]PS synthesized in the experiments in Figure 4A (total [3H]PS synthesized = [3H]PS + [3H]PE); mean ±s.d., n = 2–5 independent experiments. (B) 5x-emc cells were labeled as in Figure 4A, and the amount of radiolabeled PS and PE per OD600 was determined (mean ±s.d., n = 3 independent experiments). The data used to generate these graphs are in Table S10. (TIF) [file pbio.1001969.s002.tif]

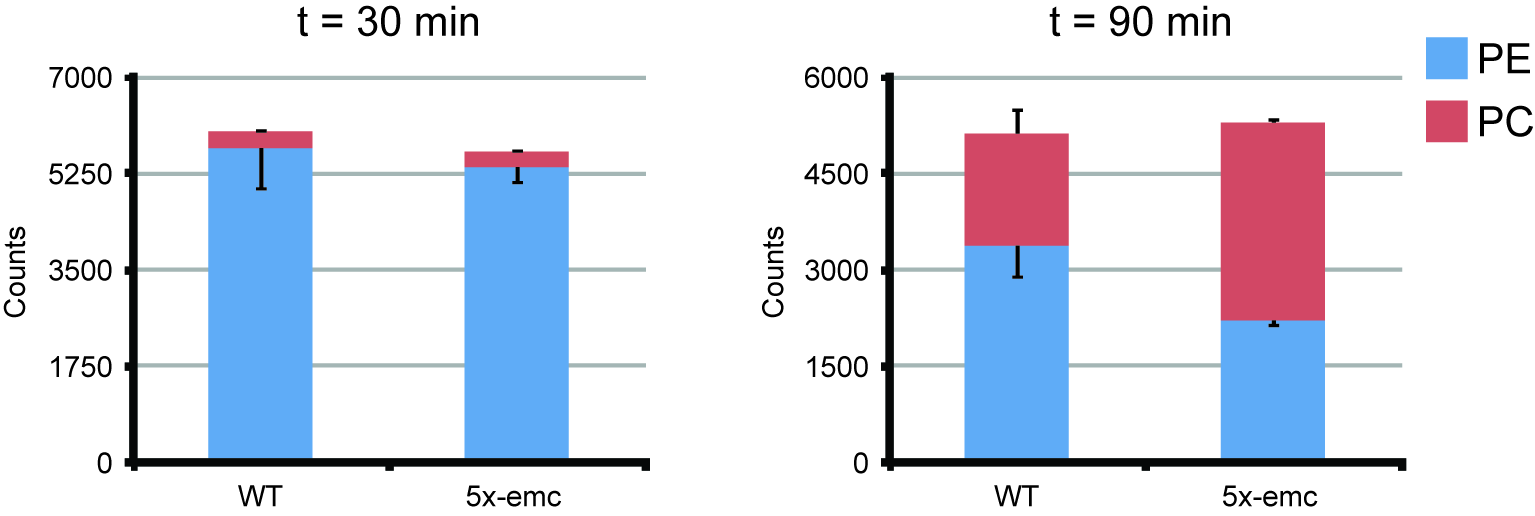

Supplement: Figure S3 — PE synthesis by the Kennedy pathway and PE methylation are not reduced in 5x-emc cells, related to Figure 4B . Wild-type and 5x-emc cells were labeled with [3H]ethanolamine for the indicated times, and lipids were extracted, separated, and quantified by HPLC and scintillation counting (mean ±s.d., n = 3). Total counts in PE and PC were not significantly different between wild-type and 5x-emc at each time point. The ∼1.7-fold increase in PC synthesized in the 5x-emc mutant at 90 min was significant (p<0.005). The data used to generate these graphs are in Table S11. (TIFF) [file pbio.1001969.s003.tiff]

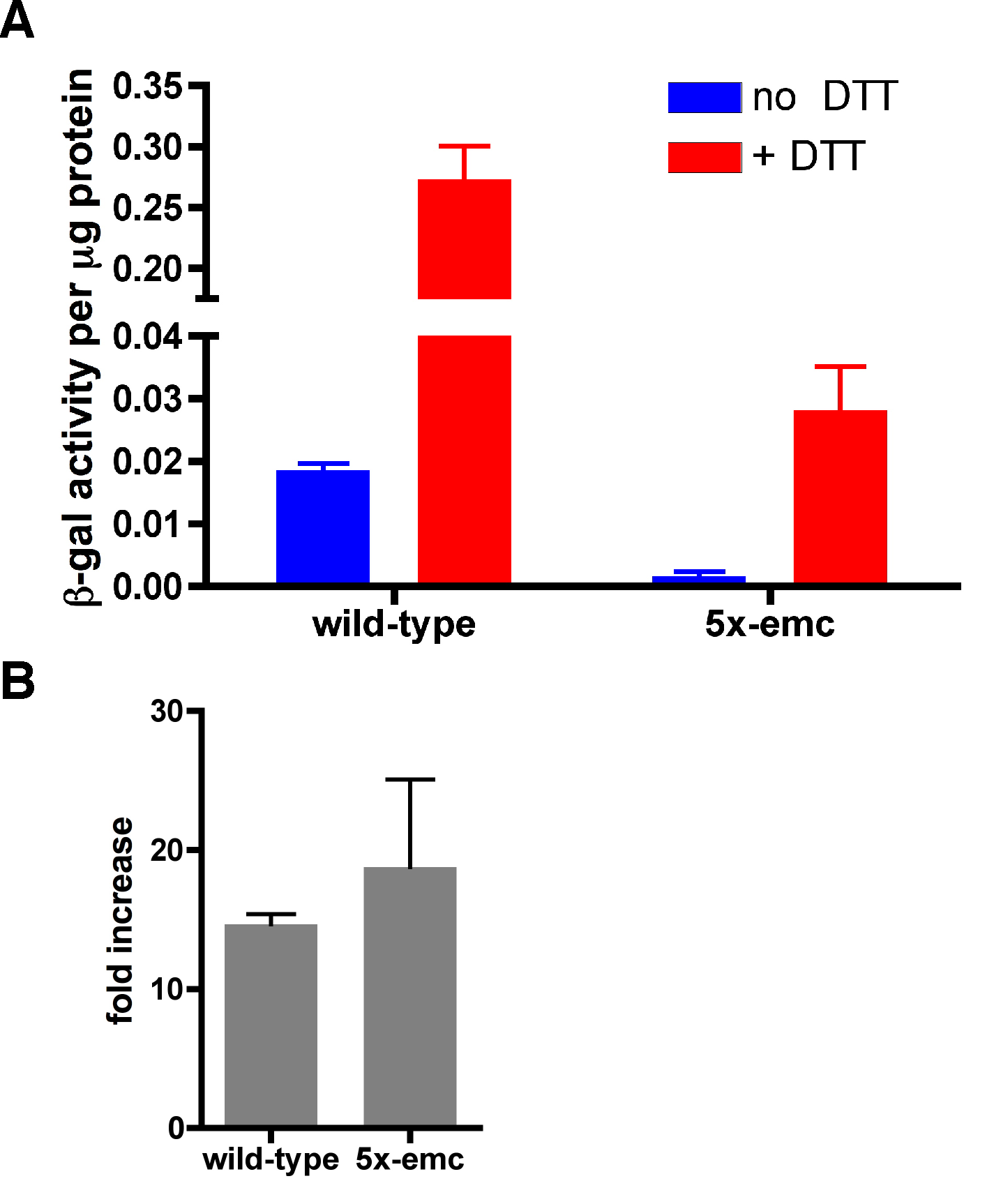

Supplement: Figure S4 — The UPR is not induced in 5x-emc cells, related to Figure 8 . Wild-type and 5x-emc cells were transformed with pMCZ-Y, a high copy plasmid carrying the lacZ gene under the KAR2 promoter. Cells were grown to mid-logarithmic growth phase in the presence or absence of 1 mM DTT for 1 h, and β-Galactosidase activity was determined (mean ±s.d., n = 3). (A) Mean β-Galactosidase activity. (B) Ratio (fold increase) of β-Galactosidase activity between cells with and without DTT. The data used to generate these graphs are in Table S12. (TIF) [file pbio.1001969.s004.tif]

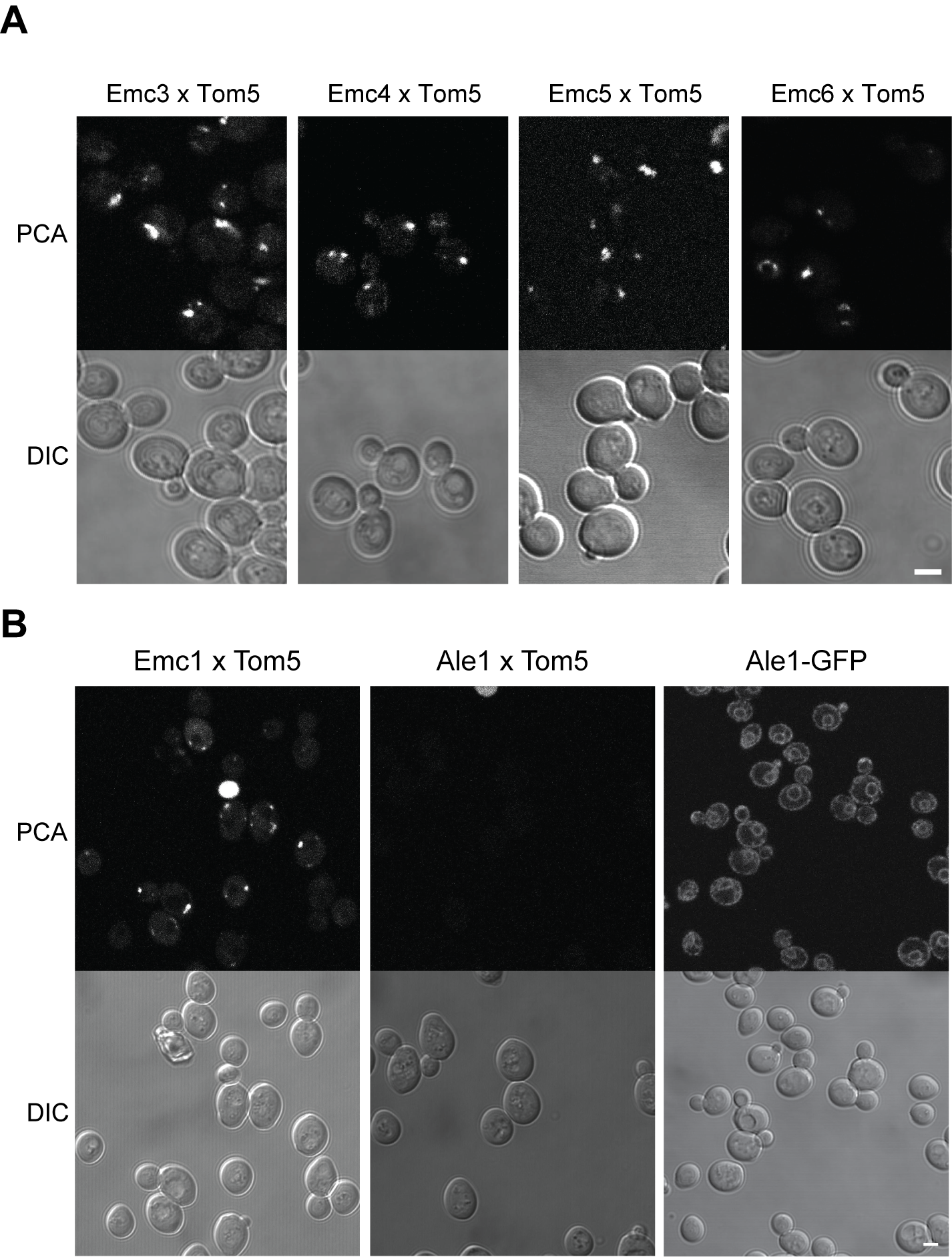

Supplement: Figure S5 — Interaction of other Emc proteins and Tom, related to Figure 9A . (A) PCA interaction of Tom5 and the indicated Emc proteins were visualized as in Figure 9A. (B) Ale1 control for PCA between the EMC and Tom5, related to Figure 7. Emc1 × Tom5 PCA (left panels) and Ale1 × Tom5 PCA (center panels) in diploids captured with identical microscope settings. Ale1 tagged at the endogenous gene locus with GFP (right panels). All scale bars, 2 µm. (TIF) [file pbio.1001969.s005.tif]

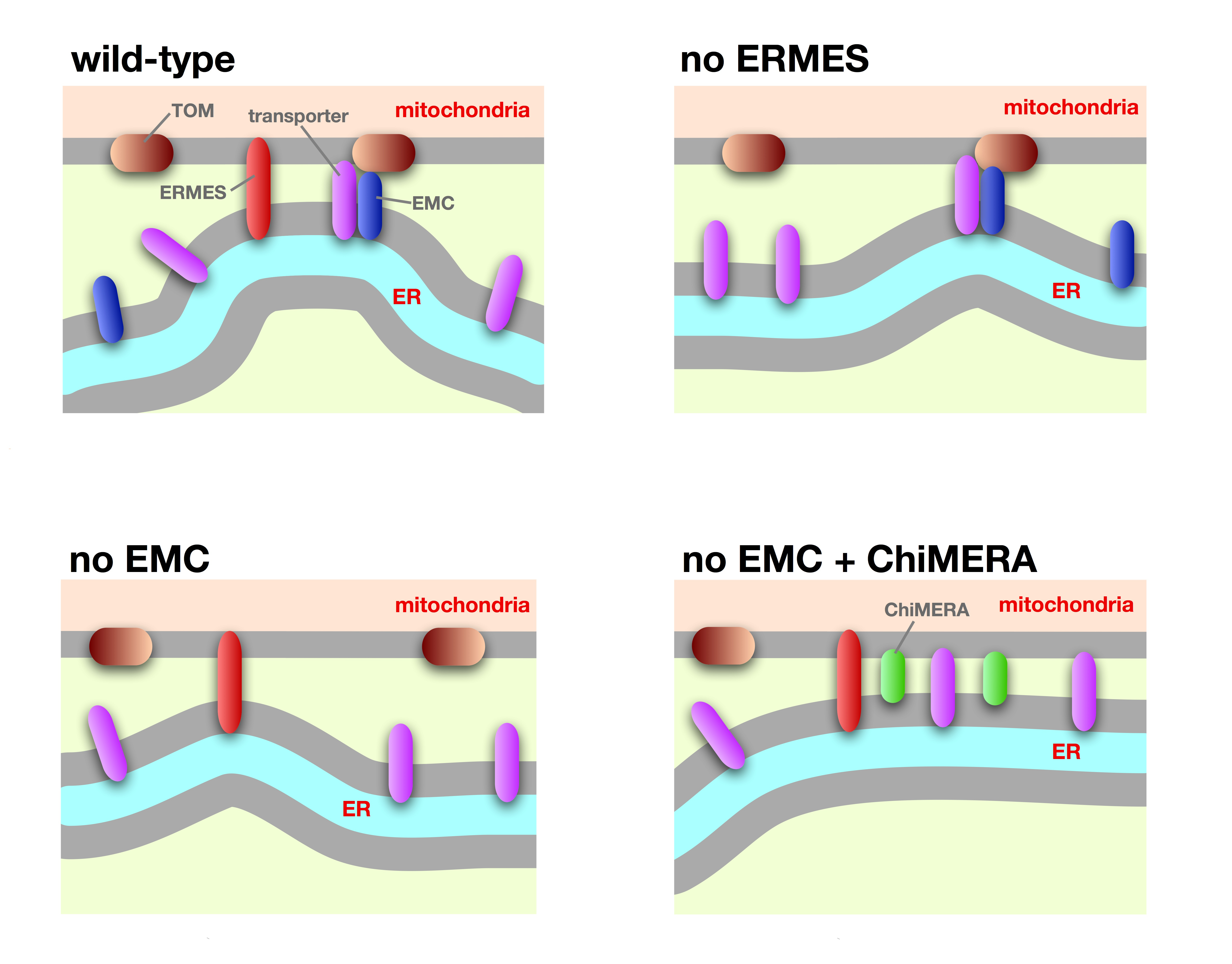

Supplement: Figure S6 — Model of the role of tethering in PS transfer from ER to mitochondria. In wild-type cells (top left), tethers are formed by both ERMES (red) and the interaction of the EMC (blue) with the TOM complex (brown). A putative transport complex (purple) associates with the EMC and facilitates PS transfer from the ER to mitochondria. In cells missing ERMES (top right), tethering is reduced but the transporter can still function at contacts mediated by the EMC. In cells missing the EMC (bottom right), the transporter is not enriched at contact sites and PS transport is reduced. When ChiMERA (green) is expressed in cells missing the EMC (bottom right), the increased tethering of the ER and mitochondria allows the PS transporter to function. (TIF) [file pbio.1001969.s006.tif]
